# Policy themes and instruments in WHO and UNICEF child food-environment guidance, 2015–2025: a computational content analysis

**DOI:** 10.3389/fnut.2026.1935052

**Published:** 2026-10-01

**Authors:** Lu Xing, Xueqi Wu

**Affiliations:** 1 School of Preschool Education, Changsha Normal University, Changsha, China; 2 School of Education, Faculty of Social Sciences and Leisure Management, Taylor’s University, Subang Jaya, Malaysia; 3 Taylor’s Culinary Institute, Faculty of Social Sciences and Leisure Management, Taylor’s University, Subang Jaya, Malaysia

**Keywords:** BERTopic, child nutrition, computational content analysis, food environments, food governance, nutritional health literacy, policy discourse, policy implementation

## Abstract

In children’s food environments, global guidance can shape policy discourse and instrument choice, but its content does not establish implementation or outcomes. In 2015–2025, the World Health Organization (WHO) and the United Nations Children’s Fund (UNICEF) published English-language guidance at the global level. A documented search, 2-author screening, and author-decision-masked expert review produced a corpus of 22 expert-refrozen documents (1,099 pages; 1,259 primary chunks). The BERTopic with document-capped discovery yielded six provisional clusters: food-marketing restrictions and children’s rights; obesity burden; nutrition-sensitive social protection and service delivery; school, physical activity, and healthy food environments; infant and young child food promotion controls; and business engagement and food-industry governance. Among 978 chunks, marketing restrictions and children’s rights accounted for 332 assigned chunks (33.9%). The expert accepted all descriptors and changed three of seven boundary decisions; applying those decisions and rerunning the full pipeline retained six identity-mapped clusters. Fixed-corpus seed and zoning diagnostics were favorable, but context expansion produced seven clusters and dominant-source removal substantially reorganized two source-concentrated clusters. To assess coding reliability, an 84-chunk exercise was archived under the earlier 19-document freeze; issuer and period profiles were descriptive. The study therefore provides a bound, auditable map rather than a stable taxonomy. Nutritional health literacy is a downstream interpretive application: no actual implementation process or literacy, behavioral, dietary-quality, or health outcome was measured.

## 1 Introduction

Children’s diets and nutrition are shaped by interacting food systems, political, social, and commercial conditions, not individual choice alone. Obesity, undernutrition, and environmental pressures share upstream drivers, including market structures and governance arrangements (1). Food environments mediate these forces through availability, affordability, vendor and product properties, marketing, convenience, and the personal resources required to acquire and consume food (2). Policy analysis must therefore attend not only to dietary recommendations but also to the rules, services, institutions, and accountability arrangements that structure children’s exposure to food-system risks.

Nutrition literacy and food literacy are related but distinct forms of health literacy, combining capacities to access, understand, evaluate, and use nutrition or food-system information (3). In children and adolescents, these capacities develop with cognitive, social, and food-related experience and are associated with diet quality, but measurement and intervention remain context-dependent (4, 5). Literacy is therefore not solely an individual educational outcome: young people may understand a recommendation yet remain constrained by affordability, availability, marketing, school provision, family resources, and institutional rules. Policy may support the development of nutritional health literacy by making reliable information accessible and may enable its application by creating environments in which informed choices are feasible. Document-level attention to each condition, however, cannot establish that the support was implemented or effective.

Food-environment governance typically requires coordinated packages of regulatory, fiscal, informational, service, and monitoring instruments (6). Food marketing is especially salient for children. Experimental evidence indicates that marketing unhealthy foods and beverages affects children’s dietary intake and preferences (7), while cross-national monitoring has documented extensive television advertising exposure across different regulatory settings (8). Reviews of policy progress have also identified incomplete implementation and the need for independent monitoring and strong accountability (9). WHO’s 2023 guideline consequently recommends comprehensive mandatory measures that reduce both exposure to and the persuasive power of harmful food marketing and frames protection from such marketing as a child-rights issue (10).

Marketing governance addresses what reaches children through commercial communication; school food environments address institutions make available, restrict, and normalize in everyday settings. School food-environment policies can improve targeted dietary behaviors, although their effects vary by policy type, implementation, and context (11, 12). Nutrition-sensitive programs may also address underlying determinants through social protection, education, agriculture, water and sanitation, and early childhood services (13). Cash-transfer evidence suggests possible improvements in diet diversity and selected child nutrition outcomes, but effects depend on program design and setting (14). Together, these literatures indicate that the relevance of a policy document to nutritional health literacy and prevention cannot be reduced to information provision: the instrument proposed, responsible actor, operational mechanism, and institutional setting also matter.

Commercial engagement creates a further governance challenge. Commercial determinants of health operate through products, corporate practices, and broader political-economic systems (15). Publicly available evidence can be used to classify and monitor food-industry political activity (16), but a document’s endorsement of partnership or business engagement should not automatically be interpreted as effective governance or adequate management of conflicts of interest. Distinguishing textual attention from implementation and effect is therefore essential.

We use a four-level analytical pathway to delimit what policy document evidence can support. Level 1 comprises policy discourse and textual commitments. Level 2 comprises the instruments proposed and the actors assigned responsibility. Level 3 comprises implementation conditions, including financing, coordination, institutional capacity, delivery arrangements, enforcement, and monitoring. These are meso-level institutional arrangements: organizational and inter-organizational mechanisms that translate macro-level guidance into practice, such as school procurement standards, service protocols, budget execution, inspection systems, and coordination across education, health, and social-protection agencies. Level 4 comprises individual and population responses: nutritional health literacy as a proximal capability potentially linked to downstream dietary behavior, diet quality, and nutritional health. This study directly observes Level 1, classifies selected Level-2 features, and records whether sampled text specifies selected Level-3 mechanisms; it does not observe actual Level-3 implementation or any Level-4 capability or outcome.

This distinction matters because implementation barriers interrupt the pathway between guidance and outcomes. Recent bibliometric evidence on stunting-reduction policy identifies insufficient funding, weak inter-organizational cooperation, cultural resistance, and inadequate multisectoral collaboration as recurring barriers, reinforcing the need to separate normative guidance from implementation capacity and outcomes (17).

Computational text analysis can organize policy corpora that are difficult to review consistently by manual reading alone. Text mining has been applied to a large national corpus of obesity-related public policies (18), but our recorded novelty search did not identify a study using this design to compare global WHO and UNICEF child food-environment guidance. Automated content analysis is not self-validating: preprocessing, corpus construction, model selection, interpretation, and external review jointly determine validity (19, 20). This problem is acute in small policy corpora, where long documents, document genre, and a few boundary decisions can dominate a model trained on many correlated text chunks.

This study addresses that gap through a reproducible computational content analysis of global-level WHO and UNICEF guidance published from 2015 to 2025. It combines document-capped clustering discovery, document-level description, two rounds of independent human coding, joint topic-label adjudication, and end-to-end corpus-boundary refits. The study asks:

1. What provisional semantic clusters organize the frozen WHO and UNICEF corpus, and how are target populations and action domains represented within those clusters?
2. How reliably can two authors classify policy instruments and implementation specificity in a topic-stratified validation sample?
3. What descriptive issuer and period contrasts appear in the primary provisional clusters, and how sensitive are those contrasts to genre, denominator, source concentration, and corpus-boundary decisions?

The aim is descriptive and methodological. Topic prevalence measures textual organization within the frozen corpus. Nutritional health literacy and prevention are downstream interpretive contexts under the four-level pathway, not outcomes evaluated in the design.

## 2 Materials and methods

### 2.1 Study design and units of analysis

We used mixed-methods computational policy content analysis to examine how global WHO and UNICEF documents organize child food-environment themes and specify policy actions. The source selection unit was the official document. The computational unit was a page- and heading-aware text chunk. BERTopic served as an exploratory organizing model, directed human coding provided substantive interpretation within a fixed validation sample, and latent Dirichlet allocation (LDA) was retained only as a supplementary sensitivity analysis. Issuer and period comparisons were aggregated to document and describe this finite corpus rather than a probability sample of all WHO or UNICEF guidance.

### 2.2 Information sources and search strategy

The search covers WHO IRIS, official WHO publication pages, and official UNICEF publication and document domains. Eligible records were English full-text documents issued or formally co-issued by WHO headquarters or UNICEF global headquarters between 1 January 2015 and 31 December 2025.

On 10 July 2026, 9 WHO IRIS query families were crossed with 3 item-type filters through the public Discover API, producing 27 recorded calls and 593 unique metadata records after deduplication. A reproducible metadata screen advanced 26 records to official full-text review. UNICEF’s internal document-search interface returned a security-verification page. We did not attempt a bypass or infer a raw count. Instead, we recorded eight searches restricted to the official unicef.org domain, a version-specific archive search, and a cross-check against UNICEF’s curated global food-systems resource index. Table 1 gives the source-specific search strategy and its reproducibility limits.

**Table 1:** Source-specific search strategy.

Source/interface	Search specification	Limits/date searched	Recorded result	Reproducibility note
WHO IRIS public Discover API	Nine exact-phrase query families crossed with publications, technical documents, and governing bodies document filters (27 calls)	English; 2015–2025; searched 10-07-2026	593 unique records after deduplication; 26 advanced to full-text review	Complete query strings, filters, counts, and API URLs are retained in the search log
UNICEF Documents and Reports internal interface	Child-population terms combined with food-environment, food-marketing, school-food, retail, policy, guidance, strategy, regulation, and program terms	English; 2015–2025; searched 10-07-2026	Security-verification page; raw result count not estimable	No bypass attempted, and no raw count claimed
UNICEF official-domain fallback	Eight documented site-restricted query families, version-specific recovery, and curated resource-index cross-check	English; 2015–2025; searched 10-07-2026	Official landing pages and PDFs verified; stable raw result count unavailable	Query strings, dates, status, and recovery notes retained
Integrated official-source verification	Record-level deduplication, version linking, landing-page verification, PDF retrieval, hashing, and text extraction	WHO/UNICEF global documents; 2015–2025	38-record candidate ledger; 35 PDFs retrieved and verified; 3 metadata/landing-only exclusions	Frozen decision ledger and source-PDF hashes preserved in the versioned freeze

The UNICEF raw result count is intentionally unreported because the official internal interface was unavailable and the fallback interface did not provide stable counts.

### 2.3 Eligibility, screening, and boundary freeze

Documents had to address children or adolescents aged 0–19 years, engage substantively with food environments or related food-system conditions, and contain policy recommendations, normative guidance, implementation guidance, accountability provisions, or explicit calls for government or food-system action. Eligible genres include global strategies, normative guidelines, action plans, program guidance, technical or policy guidance, implementation frameworks, and concise policy notes with a substantive policy function. National and regional office documents, academic articles and evidence reviews, promotional items, clinical treatment or dosage guidance, purely descriptive monitoring reports, duplicate summaries, translations, superseded editions, and inaccessible full texts were excluded (Table 2). Jointly issued documents were retained under separate issuer categories rather than attributed to WHO or UNICEF.

**Table 2:** Eligibility and corpus-construction rules.

ID	Stage	Dimension	Eligible/included	Excluded	Rationale
I01	Source	Issuer	WHO headquarters; UNICEF global headquarters; formally co-issued global document	National office or unverified issuer	Maintains a comparable global-governance level.
I02	Metadata	Date	Published from 1-1-2015 to 31-12-2025	Outside date range	Uses complete calendar years and the SDG-era policy window.
I03	Metadata	Language	English full text	Non-English-only record	Avoids translation bias and reduces manual workload.
I04	Title summary	Population	Substantive focus on children or adolescents aged 0–19 years	Adult-only or unspecified population without a substantive child section	Freezes the target population.
I05	Title summary	Domain	Addresses food environments; diets; food marketing; school food; retail; labeling; fiscal measures; affordability; food systems; or related regulatory action	Clinical treatment or nutrient-dose guidance without food-environment policy content	Aligns the corpus with Food Policy and Economics.
I06	Full text	Policy function	Contains recommendations; normative guidance; implementation guidance; accountability provisions; or calls for government/food-system action	Purely descriptive status or monitoring report	Ensures substantive policy-analysis value.
I07	Full text	Genre	Strategy; guideline; action plan; program guidance; technical note; policy guide; implementation framework	News release; promotional webpage; slide deck; conference material; academic article	Reduces genre noise.
I08	Full text	Access	Publicly accessible complete text	Abstract or summary only	Required for page-aware extraction and reproducibility.
E01	Deduplication	Version	Complete authoritative English edition	Executive summary when full report exists	Avoids duplicated content and unequal text length.
E02	Deduplication	Translation	One English version	Translated duplicate	Prevents duplicate policy content.
E03	Deduplication	Supersession	Current edition within the study period	Superseded edition with no analytical need	Prevents version overcounting; substantive editions may be retained with linkage.
E04	Full text	Document role	Core normative document	Annual results or surveillance report without policy recommendations	Separates policy guidance from organizational reporting.
E05	Full text	Clinical scope	Population or food-environment policy	Therapeutic feeding; disease treatment; dosage protocol	Avoids clinical-scope drift.
E06	Full text	Administrative level	Global headquarters	Regional office unless a protocol amendment is approved	Reduces administrative-level confounding.
E07	Full text	Genre tier	Tier A core normative	Tier B contextual report retained outside the core unless included in an explicit sensitivity analysis	Prevents unexamined mixing of normative and agenda-setting genres.

The final 22-document boundary was refrozen after the completed independent external review; the prior 19-document freeze remains archived.

The integrated candidate ledger contained 38 records. Thirty-five PDFs were retrieved and text-checked; three metadata or landing-page records remained excluded. Seven boundary cases were independently reviewed by the two manuscript authors and then adjudicated. This decision-only stage retained 19 core documents, assigned 14 records to contextual status, and excluded 5 records.

The two authors subsequently completed a fail-closed retrospective audit. Each author separately confirmed that three ambiguous amendment flags did not document or implement a rule change and attested to the same 38-record decision-ledger hash. The resulting author freeze was created on 11 July 2026 under freeze ID boundary_freeze_20260711T124423958Z. Historical decision dates that could not be recovered from a named contemporaneous source were preserved as missing, and seven added rationales were recorded as retrospective joint confirmations rather than reconstructed independent ratings.

In response to peer review, one independent professor with nutrition-policy and food-governance expertise, with no involvement in corpus or model development and no declared competing interest reviewed the same seven boundary cases with the authors’ decisions masked. The expert also reviewed six provisional cluster descriptors using organization-masked evidence. The expert accepted all six descriptors and changed three boundary decisions: P005, P021, and P036 were reassigned to the core corpus. We applied those decisions without author override, refroze the complete ledger for 22 core, 12 contextual, and 4 excluded records, and reran extraction, representation, clustering, diagnostics, document-level analysis, and figures. The expert-refrozen corpus was created on 24 August 2026 under freeze ID boundary_freeze_20260824T091443478Z and is the sole primary corpus in the revised analysis (Figure 1).

**Figure 1:**
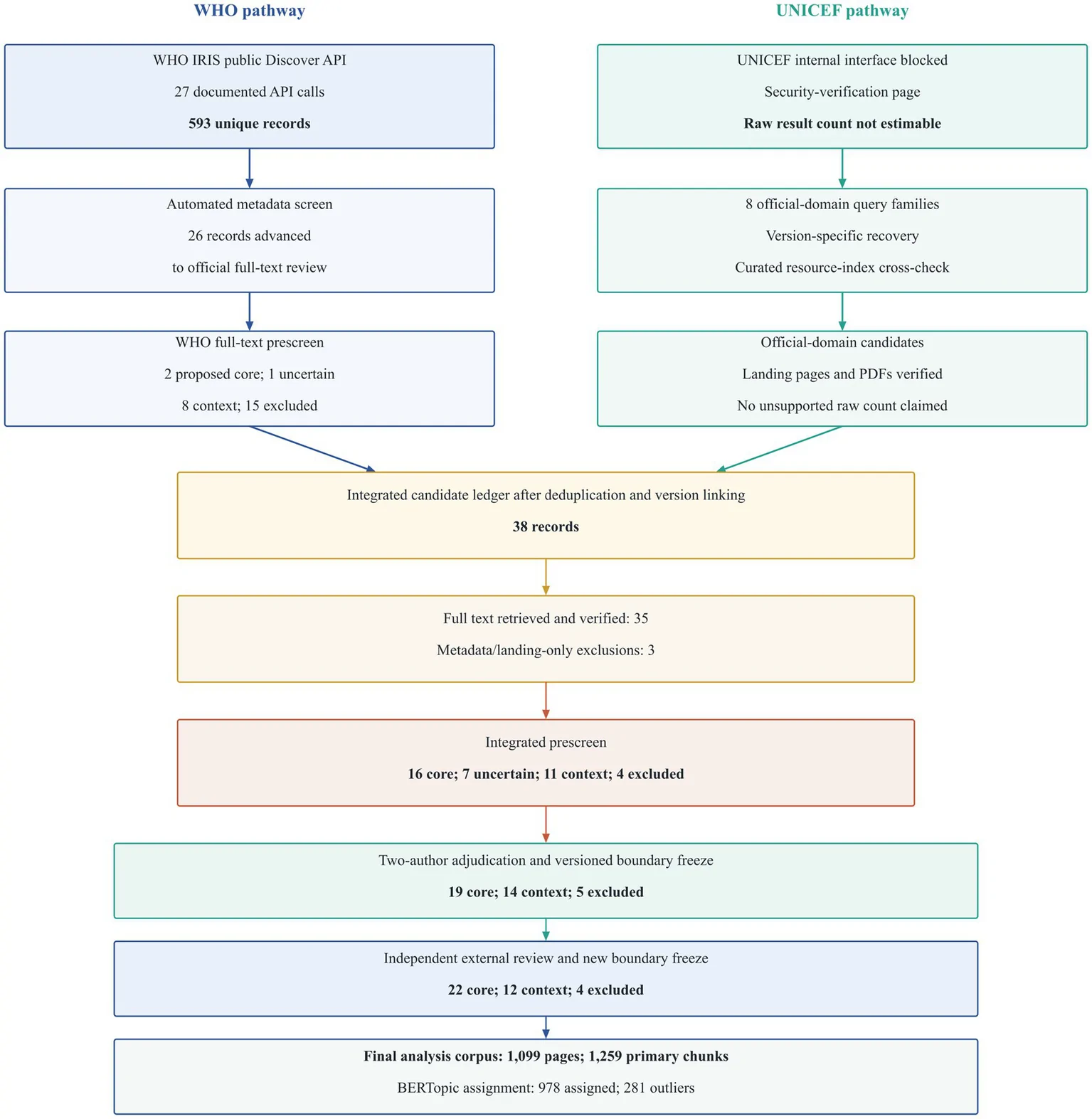
Policy document identification and screening flow.

### 2.4 Text extraction, zoning, and segmentation

Original PDFs were preserved unchanged. Embedded text was extracted page by page and audited using page counts, text yield, replacement-character checks, repeated-page-furniture detection, and source-file hashes. Rule-based zoning identified front matter, executive summaries, substantive body text, recommendations, implementation and accountability material, references, and annexes. The primary corpus retained body, recommendation, and implementation/accountability zones. The extended zoning sensitivity additionally retained executive summaries and annexes while continuing to exclude front matter and references.

Text was divided into headings, page boundaries, and sentence boundaries. Chunks targeted approximately 210 whitespace-delimited words, used a nominal maximum of 270 words, and retained self-contained units of at least 90 words; merging short adjacent units could produce chunks of up to approximately 310 words. Each chunk retained a stable identifier, source document, page span, heading, zone, issuer, period, genre, word count, and SHA-256 text hash. The expert-refrozen primary corpus contained 1,259 chunks, and the extended zoning corpus contained 1,467 chunks. Chunk counts describe computational exposure and are not treated as independent document counts.

### 2.5 BERTopic analysis

BERTopic is a modular topic-modeling framework that combines document representations, dimensionality reduction, clustering, and class-based TF-IDF topic representations (21). To keep the representation local and auditable, we used TF-IDF/SVD vectors rather than a downloaded transformer. A lowercase TF-IDF vectorizer was fitted to all 1,259 primary chunks using unigrams and bigrams, min_df = 3, max_df = 0.90, a 15,000-feature ceiling, sublinear term frequency, English stop words, and a small set of organization and publication boilerplate stop words. The resulting matrix contained 1,259 rows and 12,699 features. A 100-component truncated SVD representation was likewise fitted to the full matrix, L2-normalized, and explained 27.02% of TF-IDF variance.

UMAP/HDBSCAN clustering discovery used document-capped samples to limit unequal document exposure at the clustering stage. The cap did not make the complete representation document balanced because the TF-IDF vocabulary and SVD components had already been estimated from all chunks. An initial grid evaluated 24 UMAP/HDBSCAN configurations, followed by targeted candidates that varied the per-document cap (15, 20, or 25 chunks) and minimum cluster size. UMAP provided dimensionality reduction (22), HDBSCAN provided density-based clustering and outlier retention (23), and normalized pointwise mutual information was one component of the coherence audit (24). Selection considered coherence, topic diversity, outlier proportion, document coverage, dominant-document share, stability, and substantive interpretability.

The selected configuration, D15A, used 320 clustering-discovery chunks capped at 15 per document. Within that sample, the cap limited any one of the 22 documents to at most 4.7% of discovery observations while retaining all documents and enough observations for a minimum cluster size of 20. This parameter addressed a concrete small-corpus risk: without a cap, long documents could determine the neighborhood and density structure. Compared with the initial cap-40 grid, D15A substantially reduced mean single-document dominance while preserving coherence. Among the targeted cap-15, cap-20, and cap-25 candidates, D20A and D25A had slightly lower dominance or broader mean document coverage but produced coarser five- and three-cluster solutions with markedly lower NPMI coherence (0.146 and 0.096 versus 0.194). D15B had slightly higher coherence but a higher outlier rate, lower coverage, and higher dominance. D15A was therefore selected as a balance across coherence, diversity, outlier rate, coverage, concentration, and substantive interpretability; obtaining six clusters was not itself a selection criterion (Supplementary Table S9A). The cap balances exposure only during clustering discovery; the vocabulary and SVD representation still use the complete corpus. UMAP used n_neighbors = 15, n_components = 5, min_dist = 0.0, cosine distance, and seed 42. HDBSCAN used min_cluster_size = 20, min_samples = 5, Euclidean distance, and excess-of-mass cluster selection. Topic representation used class-based TF-IDF with unigram and bigram terms. The seed-42 model was then applied to all 1,259 primary chunks; outliers remained unassigned.

Model diagnostics included 5 random seeds, 22 leave-one-document-out refits, and a comparison of document-level prevalence under primary and extended zoning. The seed refits used the fixed 320-chunk discovery sample and the complete-corpus TF-IDF/SVD representations. Each leave-one-document-out diagnostic removed that document’s capped discovery chunks but retained TF-IDF/SVD learned from all 1,259 primary chunks; the zoning diagnostic likewise transformed the extended-zone chunks through the fixed representation and selected model. These are conditional representation diagnostics, whereas the corpus-boundary analyses in Section 2.9 rebuild the complete representation and clustering pipeline. A 12-topic candidate was retained only because of granularity sensitivity because its themes showed greater single-document concentration.

### 2.6 LDA and cross-model sensitivity

LDA (25) used unigram and bigram counts with min_df = 3, max_df = 0.90, and max_features = 15,000 on a document-balanced sample capped at 40 chunks per document. Candidate topic counts were 5, 7, 9, 11, 13, and 15. A seven-topic solution was retained for further diagnostics. Four prior settings were evaluated across 5 random seeds with a 200-iteration ceiling. Cross-model correspondence was calculated among the 978 chunks assigned by BERTopic using adjusted mutual information (AMI), normalized mutual information (NMI), adjusted Rand index (ARI), and optimal top-term matching.

### 2.7 Directed policy coding and human validation

A fixed masked sample of 84 non-outlier chunks was selected under the pre-expert 19-document freeze, with 14 chunks from each original BERTopic topic and all 19 source documents represented. No document contributed more than eight sampled chunks. Sampling covered low, middle, and high topic-membership strength and secondarily balanced issuer, period, and document coverage. Organization names were replaced with [ORGANIZATION], while the model topic, issuer, period, and the other coder’s responses were hidden. Because the expert boundary amendment added three documents after this exercise was locked, the sample was archived as a methodological-validation dataset and was not extended *post hoc* or represented as covering the 22-document corpus.

The two manuscript authors independently assigned one primary problem-frame code, one primary policy-instrument code, and one implementation-specificity level to every chunk. Policy-instrument options were regulation, fiscal measures, information/education, services/programs, partnerships/voluntary actions, monitoring/accountability, no clear instrument, and mixed instruments. Implementation specificity ranged from a normative principle (S0) to an explicit action (S1), an action with an identified responsible actor (S2), and an action with an operational mechanism such as a target, timeline, financing, enforcement, monitoring, or delivery provision (S3). S3 was deliberately compound: all three elements had to appear within the same chunk.

Both round-1 files were locked before comparison. Agreement was reported as category prevalence, exact agreement, Cohen’s kappa, and Gwet’s AC1; implementation specificity additionally used linear-weighted kappa. No numerical reliability threshold was specified before coding. A rule-based reporting gate required calibration to remove directional ambiguity, materially improve agreement, and leave a stable rule that both authors could apply independently; dimensions that failed this gate were excluded. Uncertainty intervals used 10,000 bootstrap resamples of the 19 source documents as clusters. Following round-1 ambiguity, both authors independently approved a revised rule set, recoded the problem frame for all 84 units, and recoded the implementation specificity for 66 units where at least 1 round-1 rating was S1 or S2. Eighteen S0/S0 agreements and all round-1 policy instrument ratings remain locked. Because recalibration and recoding were used in the same validation sample, round-2 agreement is reported as post-calibration reproducibility within that sample rather than as independent holdout validation.

Round 2 produced high post-calibration implementation-specificity agreement within the same sample but persistent problem-frame disagreement. Both authors approved excluding the problem frame rather than performing a third *post hoc* calibration on the same sample. The authors adjudicated 11 policy-instrument disagreements and three implementation-specificity disagreements in a separate consensus file without overwriting independent ratings.

The authors also independently reviewed the six candidate topic labels using three organization-masked excerpts from distinct documents per topic. Exact agreement was treated descriptively because only six topic-level observations were available. A joint record preserved both independent responses, resolved the original label wording and evidence-coverage ratings, and assigned each topic one of three reporting states: retain, retain with caution, or exclude from comparative interpretation. In response to peer review, the display descriptors for Clusters 3 and 5 were refined to make the leading terms and governance content explicit; a crosswalk preserved the historical locked labels and the revision-stage wording.

The independent expert then reviewed all six descriptors and accepted each one. Because that review preceded the application of the expert’s three boundary changes, descriptor transfer to the expert-refrozen solution required an explicit post-refit audit. The complete 22-document refit retained 6 clusters; optimal one-to-one matching mapped each pre-review cluster to the identically numbered refit cluster, and same-cluster top-10-term Jaccard similarity ranged from 0.667 to 1.000. All values exceeded a revision-stage operational transfer gate of 0.60. This gate was not preregistered, and the single-expert assessment does not establish definitive construct validity. The labels are therefore externally reviewed but remain provisional descriptors of a boundary-dependent model solution.

### 2.8 Document-level comparisons

BERTopic assignments were aggregated into document-level topic shares before issuer and period comparisons. The primary comparison was restricted to Topics 0, 1, and 3, which were retained without a source-coverage caution. Topics 2 and 4 were reported descriptively in separately labeled sensitivity families. Topic 5 remained in the non-outlier prevalence denominator but was omitted from all the comparative rows.

Direct issuer comparisons used 8 WHO and 11 UNICEF documents. Two WHO-UNICEF documents and one other jointly issued document remained separate and were not reassigned. Period comparisons used all 22 documents and the fixed global-topic solution for 2015–2019 versus 2020–2025. Independent period-specific models were not fitted because only seven documents were available in the earlier period.

An exact multivariate permutation diagnostic first compared the three-cluster mean vector for issuer and period; cluster-specific diagnostics followed. Named families received separate Benjamini-Hochberg adjustments. Document-mean differences and 95% document-resampling stability intervals used 10,000 percentile bootstrap replicates with seed 20,260,711. These intervals describe the dispersion under resampling of the observed documents and are not test-equivalent confidence intervals. The exchangeability condition required by permutation was not established because issuer and period were structurally associated with genre, document length, and source composition. Genre-stratified permutations addressed only the observed genre categories and could not remove the remaining dependence. Accordingly, *p* and *q* values were retained as supplementary finite-corpus diagnostics and were not used as evidence of similarity, stability, or continuity. Supplementary Tables S11A–S11C report means, differences, bootstrap intervals, cluster-specific *p*- and *q*-values, and multivariate diagnostics; the accompanying reproducibility archive preserves the complete primary and sensitivity outputs.

Three denominator and source-concentration sensitivities were evaluated for primary topics: topic counts divided by all primary chunks, shares renormalized over Topics 0–4, and the non-outlier denominator after removal of two Topic-5-dominated documents. Issuer sensitivity diagnostics are permuted labels within document genre; period sensitivities use equal-weight issuer-standardized means.

The directed-coding sample was deliberately balanced by topic and had no known corpus-level inclusion probabilities. Policy-instrument and implementation-specificity outputs were therefore restricted to the methodological validation of coding reliability and rule application. Category frequencies were not used to estimate instrument prevalence, policy priority, or implementation readiness in the corpus, and no issuer or period inference was drawn from the coded sample.

### 2.9 Corpus-boundary sensitivity

*Post hoc* full-pipeline sensitivity analyses compared the prior author-frozen 19-document corpus, the primary expert-refrozen 22-document corpus, and a 23-document context-expanded corpus that added the remaining plausible contextual record P014. For every scenario, TF-IDF and the normalized 100-component SVD were rebuilt from that scenario’s text before D15A clustering was repeated. This tested whether the thematic map survived plausible changes in which documents were treated as core evidence rather than merely applying fixed representations to altered records.

Refit topics were aligned to the 22-document primary solution through optimal one-to-one top-10-term Jaccard matching. Unmatched topics were assigned zero in the aggregate similarity score. We also performed three full-pipeline leave-dominant-source-out refits by removing P032 for Cluster 2, P017 for Cluster 4, and P031 for Cluster 5. Mapped comparisons were calculated only when a primary topic had a one-to-one match; an unmapped topic was not encoded as zero. All matching rules and similarities are descriptive, *post hoc* diagnostics rather than preregistered construct-equivalence tests.

### 2.10 Reproducibility, ethics, and AI-assisted work

Search logs, screening decisions, source URLs and hashes, extraction audits, zoning and chunk manifests, preprocessing rules, model parameters and seeds, independent coding, agreement outputs, consensus records, and the freeze manifest were maintained as separate auditable layers. Full-text redistribution depends on document licenses; the reproducibility package therefore contains metadata, hashes, code, and permitted derived data rather than source PDFs or complete chunk text.

The analysis used public organizational documents and no personal data. During manuscript preparation and revision, the authors used OpenAI Codex (desktop application, GPT-5 family; OpenAI, San Francisco, CA, United States; accessed 24 August 2026) to assist with drafting and debugging the analysis code, reproducibility and data-consistency checks, and English-language polishing and editing. No generative AI image tool was used. All five figures are deterministic, data-derived visualizations produced from audited study data by the authors’ analysis scripts, and all tables were generated from verified study outputs. No generative AI system made screening, coding, boundary, adjudication, analytical, or interpretive decisions. The authors made the author-stage decisions and reviewed all computational outputs; the independent expert made the masked boundary and descriptor judgments described in Sections 2.3 and 2.7. The authors remain responsible for the accuracy of the text, data, citations, and final submission.

## 3 Results

### 3.1 Corpus identification and characteristics

The expert-refrozen core corpus contained 22 global-level policy documents: 11 UNICEF documents, 8 WHO documents, 2 WHO-UNICEF documents, and 1 document with another joint issuer. Seven documents were published in 2015–2019 and 15 in 2020–2025. Implementation guidance was the largest genre family (11 documents), followed by policy briefs or notes (7) and high-level normative documents (4). The corpus comprises 1,099 verified pages and 1,259 primary chunks. BERTopic assigned 978 chunks to a cluster and retained 281 as outliers (22.32%). Figure 2 and Table 3 report document and chunk distributions separately.

**Figure 2:**
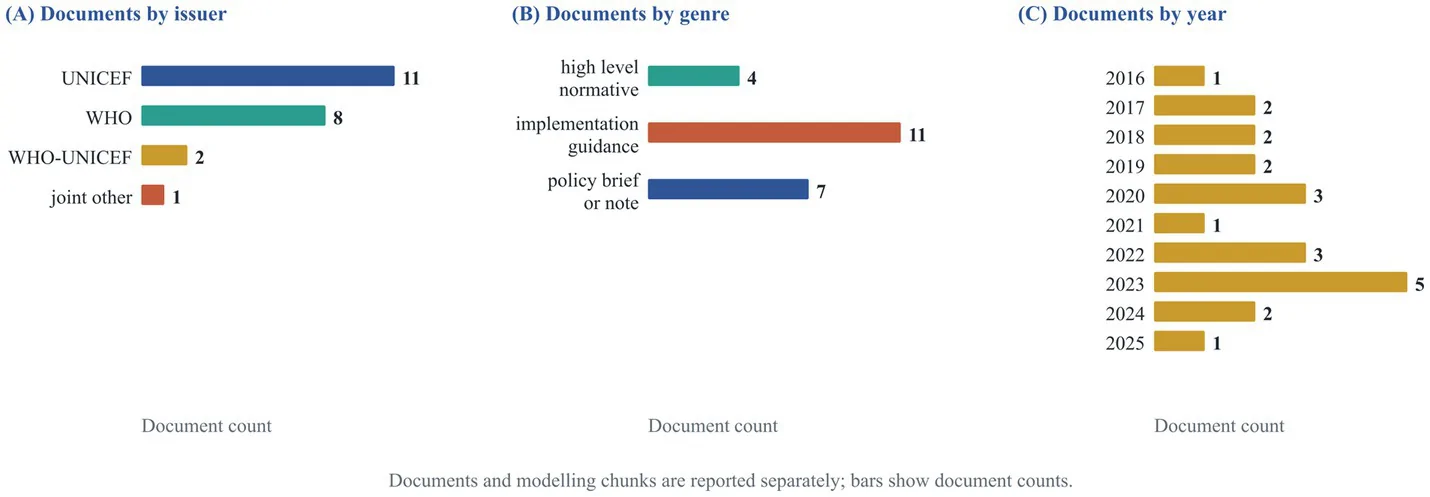
Expert-refrozen corpus distribution. Document counts by **(A)** issuer, **(B)** genre, and **(C)** publication year.

**Table 3:** Expert-refrozen core corpus characteristics.

Dimension	Category	Documents	Verified pages	Primary chunks	Topic-assigned chunks	Outlier chunks
Overall	Expert-refrozen analysis corpus	22	1,099	1,259	978	281
Issuer group	UNICEF	11	643	740	587	153
Issuer group	WHO	8	338	322	252	70
Issuer group	WHO-UNICEF	2	90	161	111	50
Issuer group	Joint other	1	28	36	28	8
Period	2015–2019	7	371	362	261	101
Period	2020–2025	15	728	897	717	180
Genre family	High-level normative	4	334	350	262	88
Genre family	Implementation guidance	11	545	632	509	123
Genre family	Policy brief or note	7	220	277	207	70

Issuer, year, period, genre, verified page count, and source PDF hash were completed for all 22 expert-refrozen core documents. Chunk counts are not independent of document counts.

### 3.2 Provisional clusters and source coverage (RQ1)

Within the expert-refrozen corpus, the model produced six clusters. Historical two-author adjudication established their reporting statuses, revision-stage wording was refined to two display descriptors, and the independent expert accepted all six descriptors (Table 4). The complete post-expert refit preserved an identity lexical mapping to the pre-review solution, with same-cluster top-10-term Jaccard values of 0.667–1.000. Because the original two-author exact agreement was only 1/6, one external expert reviewed the pre-amendment evidence, and the transfer gate was *post hoc*, these remain provisional clusters rather than independently established themes or a stable policy taxonomy.

**Table 4:** Revision-stage provisional cluster descriptors and reporting status.

Topic	Jointly adjudicated label	Assigned chunks, *n* (%)	Docs, *n*	Top five terms	Evidence/reporting status
C0	Food-marketing restrictions and children’s rights in food environments	332 (33.9%)	9	Marketing, food, food marketing, rights, children	Adequate; retain; dominant-document share: 27.4%; caution: none
C1	Childhood overweight and obesity: epidemiological burden and global trends	182 (18.6%)	18	Obesity, overweight, children, childhood, years	Adequate; retain; dominant-document share: 18.7%; caution: burden frame not policy instrument
C2	Nutrition-sensitive social protection and service delivery	179 (18.3%)	5	Nutrition, social, social protection, protection, programs	Limited; retain with caution; dominant-document share: 39.1%; caution: limited source and single-issuer coverage
C3	School, physical activity, and healthy food environments	84 (8.6%)	10	Physical, physical activity, activity, foods, healthy	Adequate; retain; dominant-document share: 22.6%; caution: combined school food and physical activity domains
C4	Infant and young-child food-promotion controls	83 (8.5%)	8	Digital, infants, young, marketing, promotion	Mixed; retain with caution; dominant-document share: 48.2%; caution: near half single-document concentration
C5	Business engagement and food-industry governance	118 (12.1%)	5	Business, industry, engagement, upf, nutrition	Limited; inventory only; exclude from comparisons; dominant-document share: 78.0%; caution: high single-document concentration

Percentages use the 978 non-outlier chunks as the denominator. The independent expert accepted all six descriptors; post-refit transfer required identity mapping and a minimum observed same-topic top-10 Jaccard of 0.667. Topic 5 remains in the inventory but is excluded from comparative interpretation.

Cluster 0, Food-marketing restrictions and children’s rights in food environments, was the largest assigned cluster, with 332 of 978 non-outlier chunks (33.9%) across 9 documents. Cluster 1, Childhood overweight and obesity: epidemiological burden and global trends, contained 182 chunks (18.6%) and had the widest document coverage (18 documents); it describes burden rather than a policy instrument. Cluster 2, Nutrition-sensitive social protection and service delivery, contained 179 chunks (18.3%) across 5 documents and was retained with caution because of limited source and issuer coverage. Cluster 3, School, physical activity, and healthy food environments, contained 84 chunks (8.6%) across 10 documents; this wording retains prominent physical activity terms and excerpt content. Cluster 4, Infant and young-child food-promotion controls, contained 83 chunks (8.5%) across 8 documents and was retained with caution because one document contributed 48.2% of its assigned chunks.

Cluster 5, Business engagement and food-industry governance, contained 118 chunks (12.1%) across 5 documents, but 78.0% came from UNICEF’s 2022 *Engaging Business for Maternal and Child Nutrition: UNICEF Programming Guidance*. Its representative passages concern private-sector roles, due diligence, risk management, safeguards, and circumstances in which direct engagement may be inappropriate. The cluster therefore captures source-dependent programming discourse rather than a UNICEF-wide position. It was retained in the inventory for transparency and excluded from issuer, period, and policy-implication comparisons.

The primary issuer description was restricted to Clusters 0, 1, and 3 (Figure 3). Mean Cluster 0 share was 12.7% for WHO and 19.8% for UNICEF; Cluster 1 means were 28.3 and 21.4%; and Cluster 3 means were 21.6 and 12.5%, respectively. The group sizes were 8 WHO and 11 UNICEF documents; the resampling intervals were wide, and the genre was unevenly distributed. Denominator, source-concentration, and genre-stratified checks did not establish a stable issuer contrast. Effect sizes, document-level distributions, and all exploratory permutation outputs are reported in the Supplementary material; non-significant diagnostics are not interpreted as evidence of similarity.

**Figure 3:**
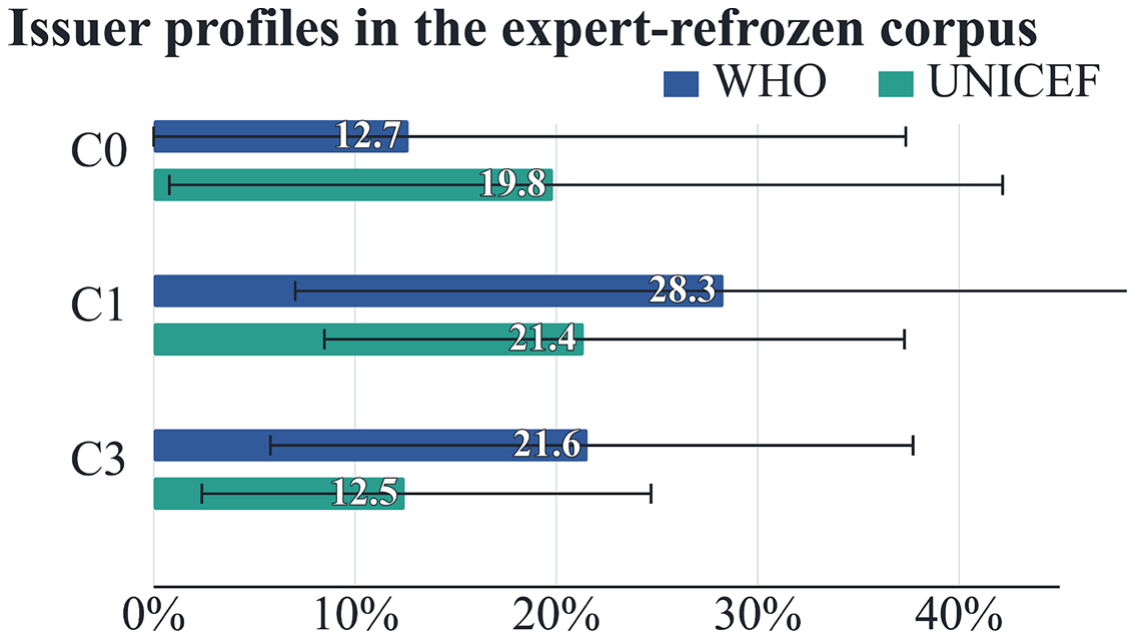
Descriptive issuer profiles for the three primary provisional clusters in the expert-refrozen corpus.

### 3.3 Methodological validation of human coding (RQ2)

Policy-instrument coding achieved high agreement in round 1 (exact agreement = 0.869; Cohen’s kappa = 0.839; Gwet’s AC1 = 0.852). After rule-based recalibration, implementation-specificity coding showed high post-calibration agreement within the same validation sample (exact agreement = 0.964; kappa = 0.946; AC1 = 0.954); it was not an independent holdout validation. Problem-frame coding remained unreliable after recalibration (exact agreement = 0.488; kappa = 0.389; AC1 = 0.423) and was excluded. Figure 4 shows these reliability results rather than instrument-frequency comparisons.

**Figure 4:**
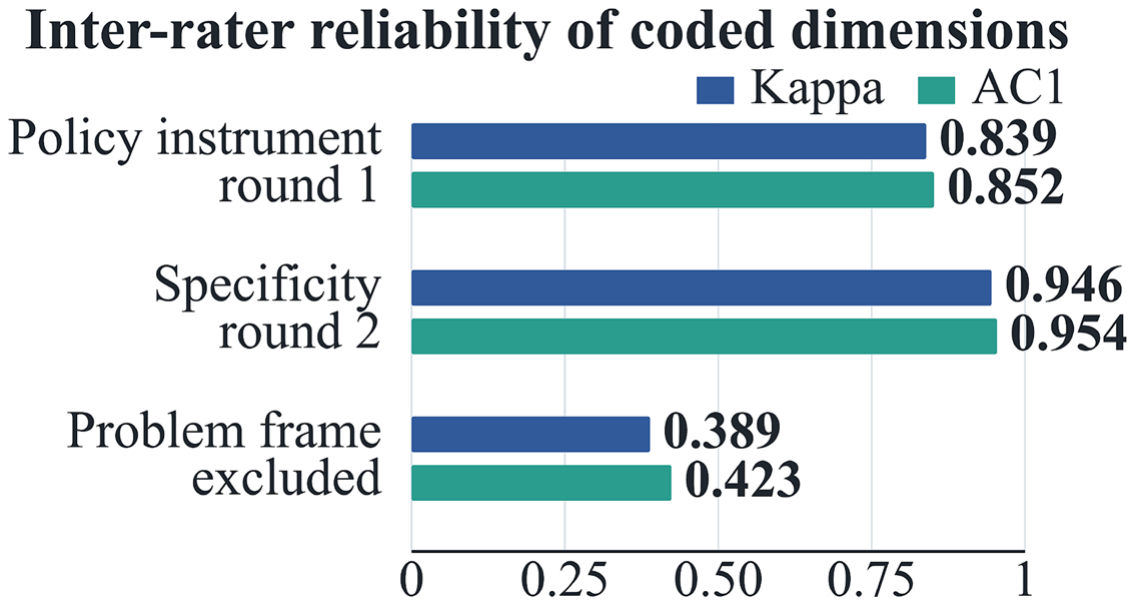
Inter-rater reliability across the two human-coding rounds.

The exercise also shows how unit choice and compound coding rules constrain interpretation. The strict S3 category was unused in this deliberately balanced passage sample, but that code behavior may reflect the within-chunk threshold, segmentation across passages, sampled passage types, or document genres. It provides no evidence that complete source documents or organizations lack financing, enforcement, timelines, delivery arrangements, or monitoring. Category distributions, confusion matrices, disagreements, revision rules, and adjudication records are provided in the Supplementary material solely as methodological audit evidence and are not used to infer corpus-wide policy priorities or implementation readiness.

### 3.4 Descriptive issuer and period contrasts (RQ3)

The primary period description used Clusters 0, 1, and 3. The mean Cluster 0 share was 24.0% in 2015–2019 and 27.9% in 2020–2025; Cluster 1 changed from 35.2 to 16.2%, and Cluster 3 from 26.5 to 8.5%. Only seven documents represented 2015–2019, compared with 15 in 2020–2025, and genre and issuer composition differed between periods. Issuer-standardized, denominator, and source-concentration checks did not establish a stable period contrast. Detailed effect sizes, document distributions, stability intervals, and exploratory permutation diagnostics are reported in the Supplementary material.

Cluster 2 appeared in only five documents and remained caution-qualified. Cluster 4 was also descriptive only because of its source concentration. We do not interpret these profiles as emergence, decline, continuity, or event-driven change. The topic-stratified human-coding sample was not used for period inference.

### 3.5 Model and human-validation audit

For the expert-refrozen corpus, the six-cluster BERTopic model showed high multi-seed assignment agreement (mean pairwise AMI = 0.8966), mean matched top-term Jaccard similarity of 0.9010, mean leave-one-document-out term Jaccard similarity of 0.8274, and low primary-versus-extended-zone prevalence divergence (mean Jensen–Shannon distance = 0.0177). The seed and leave-one-document-out refits retained the complete-corpus representation, and the zoning check used the fixed selected model; these diagnostics therefore indicate repeatability conditional on the same corpus and representation. They do not validate the cluster labels or demonstrate robustness to source selection. The supplementary seven-topic LDA model failed the convergence and stability gate: all 20 tested runs reached the 200-iteration ceiling, the mean seed AMI was 0.3593, and the mean seed term Jaccard similarity was 0.4208. Cross-model correspondence was limited (AMI = 0.3897; NMI = 0.3953; ARI = 0.2316; matched-term Jaccard = 0.3137).

The independent topic-label decision agreement of 1/6 was followed by documented joint adjudication yielding three retained clusters, two caution-qualified clusters, and one comparison-excluded cluster. The external expert accepted all six descriptors, but this single assessment and the *post hoc* transfer audit narrowed uncertainty without establishing definitive semantic validity (Table 5).

**Table 5:** Model stability and human validation audit.

Audit component	Scope/configuration	Key result	Reporting decision
BERTopic selected model	D15A; full-corpus TF-IDF/SVD representation; 320 document-capped clustering-discovery chunks; seed 42; UMAP 15 neighbors/5 components; HDBSCAN minimum cluster size 20	Six topics; discovery outliers 10.3%; full-corpus outliers 22.3%	Primary descriptive topic solution
BERTopic stability	Five seeds on a fixed cap-15 discovery sample; leave-one-document-out refits retaining complete-corpus TF-IDF/SVD; fixed-model primary-versus-extended zoning	Mean seed AMI = 0.897; seed term Jaccard = 0.901; leave-one-out term Jaccard = 0.827; mean JS distance = 0.018	Conditional representation diagnostics; not evidence of boundary invariance
LDA sensitivity	7 topics; 40 chunks per document; 20 runs at 200 iterations	Mean seed AMI = 0.359; seed term Jaccard = 0.421; all runs reached iteration ceiling	Failed convergence/stability gate; sensitivity only
Cross-model correspondence	978 BERTopic-assigned chunks compared with 7-topic LDA	AMI = 0.390; NMI = 0.395; ARI = 0.232; matched-term Jaccard = 0.314	Limited triangulation; no independent validation claim
Round 1: problem frame	Two masked coders; 84 chunks	*n* = 84; exact agreement = 0.464; Cohen kappa = 0.317; Gwet AC1 = 0.401	Recalibrated and recoded
Round 2: problem frame	Two masked coders; all 84 chunks	*n* = 84; exact agreement = 0.488; Cohen kappa = 0.389; Gwet AC1 = 0.423	Persistently unreliable; excluded by two-author consensus
Policy instrument coding	Round 1 retained; archived 84-chunk sample selected under the pre-expert 19-document freeze	*n* = 84; exact agreement = 0.869; Cohen kappa = 0.839; Gwet AC1 = 0.852	Retained; 11 disagreements adjudicated
Implementation-specificity coding	Round 2; archived 84-chunk sample selected under the pre-expert 19-document freeze	*n* = 84; exact agreement = 0.964; Cohen kappa = 0.946; Gwet AC1 = 0.954	Retained; 3 disagreements adjudicated
Topic-label review	Two independent reviewers; 6 candidate labels; joint adjudication	Independent exact label-decision agreement 1/6; consensus: 3 retain, 2 caution, 1 comparison-excluded	Negotiated interpretation disclosed; no independent validation claim
Independent external expert	One nutrition-policy/food-governance expert; author-decision-masked review of seven boundary cases and six descriptors	Three boundary changes; all six descriptors accepted; post-refit identity mapping; minimum same-topic top-10 Jaccard = 0.667	Applied P005, P021, and P036 changes; complete pipeline refitted; descriptors retained with transfer audit
Corpus boundary refits	Full text-to-TF-IDF/SVD-to-clustering refits for prior 19-document, expert-refrozen 22-document, and context-expanded 23-document corpora	Topic counts 6/6/7; penalized matched top-10 Jaccard 0.854/1.000/0.732 relative to the expert-refrozen baseline	Boundary-sensitive; the expert-refrozen 22-document solution is primary
Dominant source refits	Full-pipeline leave-dominant-document-out refits for Clusters 2, 4, and 5	Without P032: 6 topics and C2 Jaccard 0.538; without P017: 3 topics and C4 Jaccard 0.429; without P031: 5 topics and no one-to-one C5 match	Clusters 2, 4, and especially 5 remain caution-qualified; no stable-taxonomy claim

Agreement statistics describe the archived fixed 84-chunk validation set selected under the pre-expert freeze. Full-pipeline boundary and dominant-source refits are post hoc descriptive diagnostics.

The post-expert full-pipeline sensitivity results are summarized in Figure 5. The prior 19-document author freeze produced 6 clusters and penalized top-10-term Jaccard similarity of 0.854 to the expert-refrozen baseline; the primary 22-document refit produced 6 clusters; and adding the remaining contextual record produced seven clusters with similarity of 0.732. Dominant-source removal was more consequential for specific clusters. Removing P032 retained six clusters but mapped Cluster 2 at Jaccard 0.538. Removing P017 reduced the solution to three clusters and mapped Cluster 4 at 0.429. Removing P031 produced five clusters and no one-to-one match for Cluster 5. Thus, the overall six-cluster organization survived the expert’s three amendments, but the context-expanded solution and the source-dependent Clusters 4 and 5 remained structurally sensitive.

**Figure 5:**
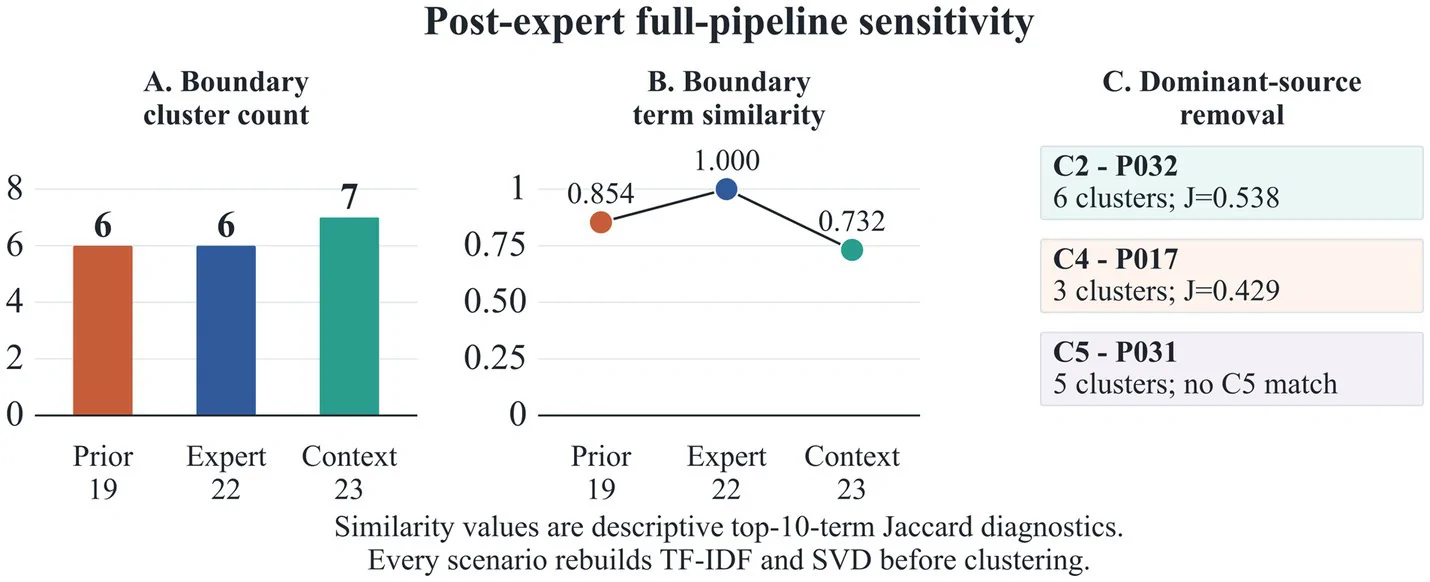
Post-expert full-pipeline sensitivity analysis. **(A)** Cluster counts across corpus-boundary refits; **(B)** top-10-term Jaccard similarity to the expert-refrozen baseline; and **(C)** leave-dominant-source-out refits for Clusters 2, 4, and 5.

## 4 Discussion

### 4.1 Principal findings

Within the expert-refrozen 22-document corpus, BERTopic produced 6 provisional clusters. Food-marketing restrictions and children’s rights formed the largest cluster. The remaining clusters describe epidemiological burden, nutrition-sensitive social protection and service delivery, school food and physical activity environments, infant and young-child promotion controls, and business engagement. Cluster 1 is a burden-description cluster rather than a policy theme, and Cluster 5 is predominantly a single-document discourse. The independent experts accept all six descriptors, but the labels remain conditional on this small corpus and model solution. These qualifications are part of the result rather than secondary caveats.

Issuer and period profiles showed descriptive differences, but the design did not support organization-wide rankings, temporal trends, equivalence, or continuity. Document-resampling intervals were wide, group sizes were small, genres were unevenly distributed, and full-pipeline boundary refits changed topic count and selected contrast directions. The defensible contribution is an auditable map of policy language and a transparent account of where comparative interpretation failed.

### 4.2 Structural conditions relevant to nutritional health literacy

The prominence of marketing restrictions and children’s rights is substantively consistent with evidence that unhealthy-food marketing influences children’s intake and preferences (7), remains widespread across countries (8), and has not been adequately controlled through existing policy and self-regulatory arrangements (9). WHO’s rights-based guideline further supports the interpretation that exposure reduction and persuasive-power restrictions belong to the same protective policy domain (10). The topic model does not independently verify these external effects, but the literature supports the policy relevance of the textual emphasis observed in the corpus.

The topic-stratified coding exercise was designed to assess coding agreement and interpretive feasibility, not to estimate the composition of the guidance portfolio. Its raw instrument frequencies therefore cannot support the claim that education is secondary across WHO or UNICEF documents. The appropriate link to nutritional health literacy is conceptual: nutrition and food literacy include functional, interactive, and critical capacities (3), while children’s ability to apply those capacities develops over time and is conditioned by social and food-system settings (4, 5).

The direct corpus finding is that several provisional clusters concern regulatory, environmental, social-protection, and service domains rather than information alone. External literature gives these domains practical relevance: coordinated food policies can alter the environments in which choices are made (6); school food-environment interventions can improve selected dietary behaviors, with heterogeneous implementation and outcomes (11, 12); and nutrition-sensitive programs and cash transfers can address underlying constraints, depending on program design and context (13, 14). On that external basis, we propose, rather than empirically demonstrate, that nutritional health literacy initiatives should be examined alongside labeling and communication, school and community food environments, marketing protection, accessible services, and accountability arrangements.

Cluster 5 requires a different interpretation. Its dominant source is a UNICEF programming guide on engaging businesses for maternal and child nutrition, including due diligence, safeguards, risk management, and conditions for declining engagement. Retaining the cluster preserves that qualitative nuance; excluding it from comparisons prevents one’s guide from being mistaken for a general organizational position. External literature similarly cautions that commercial determinants operate through products, business practices, and political-economic power (15), and that food-industry political activity requires explicit monitoring rather than the assumption that engagement itself is accountable to governance (16).

### 4.3 From textual commitment to implementation

The four-level pathway clarifies the evidentiary boundary. Topic prevalence describes Level-1 discourse, while the coding exercise tests whether selected Level-2 features and textual references relevant to Level 3 could be classified consistently in a balanced validation sample. The S0–S3 scale does not observe the Level 3 processes through which organizations finance, coordinate, deliver, enforce, or monitor action. It also provides no Level-4 evidence about literacy, behavior, diet quality, or health.

This distinction matters because policy content does not determine implementation by itself. Actors, processes, and context shape whether formal policy intentions are adopted and enacted (26), and implementation frameworks distinguish proposed actions from determinants, strategies, processes, and observed outcomes (27). The implementation barriers are identified by Azwar et al. (17), including funding, inter-organizational cooperation, cultural resistance, and multisectoral coordination, are examples of Level 3 conditions that the present corpus map cannot measure. Because the strict S3 category was unused only in a non-probability passage sample, we make no document-level inference from that observation and do not treat it as evidence of implementation readiness or its absence.

### 4.4 Temporal patterns and the limits of period comparison

The later-period corpus contained a descriptive concentration of the caution-qualified social-protection cluster and lower mean shares for two of the three primary clusters. These are observed profiles, not evidence of historical change. Only 7 documents represented 2015–2019, compared with 15 in 2020–2025, and issuer and genre composition differed between these periods. A single global model was used because independent period-specific models would not have provided a defensible basis for claims that clusters emerged or disappeared.

Accordingly, the findings do not support continuity, rebalancing, or event-driven change. The design cannot attribute any difference to the COVID-19 pandemic, a particular WHO guideline, a UNICEF strategy, or another milestone. Such claims would require a larger longitudinal corpus, pre-specified interruption points, more comparable document genres, and policy-process evidence beyond text prevalence.

### 4.5 Methodological implications for small policy corpora

Several diagnostics initially appeared reassuring. The selected BERTopic solution had high multi-seed AMI, relatively high matched-term stability, moderate leave-one-document-out similarity, and low sensitivity to the inclusion of executive summaries and annexes. However, these diagnostics answered narrower questions than boundary refits. They showed that clustering was reasonably repeatable for a fixed corpus and representation, not that the substantive taxonomy was invariant to source selection.

The post-expert 6/6/7-cluster prior/expert/context results and the dominant-source refits are therefore central. They show that the broad six-cluster organization survived the three expert amendments, but adding one remaining contextual record changed the topic count and removing individual dominant sources reorganized the solution for Clusters 4 and 5. A non-significant comparison or high fixed-corpus stability diagnostic cannot be treated as evidence of taxonomy robustness or equivalence. This finding extends general warnings that automated text analysis requires design-specific validation (19, 20): in small policy corpora, corpus-boundary and source-concentration decisions should be audited by rebuilding the entire representation and clustering pipeline, not only by applying a fixed model to alternative records.

The human validation results provide a second methodological lesson. Policy-instrument coding showed high agreement in round 1, and implementation specificity showed high post-calibration agreement within the same validation sample. Problem frames remained systematically ambiguous and were excluded rather than repeatedly recalibrated on the same sample. Topic-label agreement was only 1/6 before adjudication, even though conditional clustering diagnostics were stronger. The later independent expert review accepted the six descriptors and changed three boundary decisions. Applying those changes and rerunning the complete pipeline strengthened the audit trail; nevertheless, one expert and a *post hoc* lexical transfer rule cannot convert provisional clusters into independently established constructs.

### 4.6 Strengths and limitations

Strengths include a reproducible WHO API search, an explicit UNICEF fallback record, source-PDF hashing, page-aware extraction, document-capped clustering discovery, outlier retention, document-level aggregation, two rounds of masked coding, separate consensus records, multiplicity control, and full-pipeline boundary refits. Negative evidence was preserved: the LDA convergence gate failed, problem-frame calibration failed, independent topic-label agreement was low, and boundary invariance was not established.

The main limitation is corpus coverage. The study is English-only and restricted to global-level WHO and UNICEF documents. Regional and national documents were excluded to keep issuer, administrative level, and policy function more comparable; consequently, the corpus cannot support recommendations about how schools, communities, or countries implement global guidance. The UNICEF internal interface was unavailable, so the official-domain fallback cannot provide a reproducible raw-result denominator and may have missed eligible documents. The 22-document corpus is too small for strong issuer or period inference, and document genres are unevenly distributed. The initial boundary audit was completed retrospectively: final decisions and ledger hashes are frozen, but original independent reasons and some decision dates could not be recovered from contemporaneous records. Added author rationales are joint retrospective confirmations. The later expert review was independent and author-decision-masked but involved only one expert; the six descriptors were reviewed before the boundary amendments were applied and were transferred to the refit solution using a non-preregistered operational lexical gate.

Textual prevalence is not policy commitment, adoption, financing, implementation capacity, enforcement, or effectiveness. Chunk counts are correlated within documents, and document-level resampling cannot create information absent from a small fixed corpus. Clusters 2 and 4 have limited or concentrated source coverage, and Cluster 5 cannot support comparative interpretation. The 84-chunk human sample was selected under the pre-expert 19-document freeze, balanced by cluster, and is unsuitable for estimating corpus-wide policy-instrument prevalence in either the early or expert-refrozen corpus. Its compound S3 code cannot determine which operational components are absent across whole documents. The LDA model failed its stability gate, cross-model correspondence was limited, initial independent label agreement was low, and the BERTopic solution remained sensitive to context expansion and dominant-source removal. These limitations prevented claims of institutional ranking, temporal trends, implementation readiness, or causal policy effect.

### 4.7 Future research and bounded policy implications

For policy researchers, the direct methodological implication is a two-layer workflow. Computational models can organize a corpus and identify candidate clusters, but substantive claims should then pass document-level aggregation, human coding, source-concentration checks, independent domain review, and explicit reporting gates. A cluster should not enter into institutional or temporal comparison solely because it is coherent or large.

For nutritional health literacy, the provisional map identifies policy domains to test in future implementation research: information provision, marketing control, rights, school food and activity environments, service delivery, social protection, and accountability. Any recommendation to combine these domains is a normative proposition supported by external policy and intervention literature, not an effectiveness finding from the topic model.

Future comparative studies should enlarge the document base to regional and national policy instruments while retaining administrative level, issuer, and genre as explicit strata. Global text maps could be linked to the WHO Global database on the Implementation of Food and Nutrition Action and the World Cancer Research Fund NOURISHING policy database (28, 29). Complementary indicators should include formal adoption and implementation status; budget allocation and execution; responsible agencies and coordination mechanisms; program reach; procurement or service-delivery arrangements; inspection, sanctions, and compliance; monitoring and public reporting; and downstream literacy scores, marketing exposure, dietary behavior, diet quality, and health outcomes. A prospective protocol with independently timestamped screening reasons and independent boundary review would remove the historical audit limitation encountered here.

## 5 Conclusion

Within the expert-refrozen WHO and UNICEF corpus, BERTopic produced six provisional clusters led by food-marketing restrictions and children’s rights and complemented by burden description, social protection and services, school food and physical activity environments, promotion controls for infants and young children, and source-concentrated business-engagement discourse. An independent expert accepted the 6 descriptors and changed three boundary decisions; the complete 22-document refit preserved 6 identity-mapped clusters. Archived human coding showed high agreement for the retained instrument dimension and high post-calibration agreement for implementation specificity within the same validation sample, but it does not estimate corpus-wide policy priorities. Issuer and period profiles remained descriptive, while context expansion and dominant-source refits showed residual structural sensitivity.

The study therefore offers a bounded descriptive map rather than a stable comparative taxonomy. Its relevance to nutritional health literacy is interpretive: the corpus map identifies policy domains that future studies can connect to implementation and outcomes, but it does not show that any policy package improves literacy, behavior, diet quality, or health. Methodologically, small policy-document topic models require full audit trails, document-level analysis, source-concentration restrictions, external expert review, and end-to-end boundary refitting before substantive comparisons are reported.
