# Supplementary material for: Policy themes and instruments in WHO and UNICEF child food-environment guidance, 2015–2025: a computational content analysis
Source: Front Nutr. 2026 Oct 1;13:1935052. doi: 10.3389/fnut.2026.1935052 (PMC13668553; doi:10.3389/fnut.2026.1935052)
Supplement: Supplementary file 1 [file Table_1.DOCX]

***Supplementary Material***

Scope of this revision. This supplement exposes the complete search log, candidate-record ledger, document-level cluster distribution, topic-label evidence, human-coding audit, and boundary-sensitivity diagnostics and independent domain review. The corpus, clusters, and coding sample are bounded descriptive objects; none measures nutritional health literacy, implementation, behaviour, dietary quality, or health outcomes.

# 1 S1. Search procedures and dates

WHO discovery used the public WHO IRIS Discover API. The UNICEF internal interface returned a security-verification page; no bypass was attempted and no raw-result denominator is claimed. The documented fallback used eight official-domain query families, version-specific recovery, and curated resource-index checks. Searches were conducted on 10 July 2026 for documents dated 2015-2025.

**Supplementary Table S1. Complete source-specific query log**

| **Search ID** | **Source** | **Exact query** | **Filters/window** | **Search date** | **Raw records** | **Status** |
| --- | --- | --- | --- | --- | --- | --- |
| WHO_IRIS_Q01_PUBLICATIONS | WHO IRIS public Discover API | "food environment" AND (child* OR adolescen* OR infant*) | 2015-2025; Publications | 2026-07-10 | 85 | completed |
| WHO_IRIS_Q01_TECHNICAL_DOCUMENTS | WHO IRIS public Discover API | "food environment" AND (child* OR adolescen* OR infant*) | 2015-2025; Technical Documents | 2026-07-10 | 41 | completed |
| WHO_IRIS_Q01_GOVERNING_BODIES_DOCUMENTS | WHO IRIS public Discover API | "food environment" AND (child* OR adolescen* OR infant*) | 2015-2025; Governing Bodies documents | 2026-07-10 | 11 | completed |
| WHO_IRIS_Q02_PUBLICATIONS | WHO IRIS public Discover API | "food marketing" AND (child* OR adolescen* OR infant*) | 2015-2025; Publications | 2026-07-10 | 79 | completed |
| WHO_IRIS_Q02_TECHNICAL_DOCUMENTS | WHO IRIS public Discover API | "food marketing" AND (child* OR adolescen* OR infant*) | 2015-2025; Technical Documents | 2026-07-10 | 16 | completed |
| WHO_IRIS_Q02_GOVERNING_BODIES_DOCUMENTS | WHO IRIS public Discover API | "food marketing" AND (child* OR adolescen* OR infant*) | 2015-2025; Governing Bodies documents | 2026-07-10 | 19 | completed |
| WHO_IRIS_Q03_PUBLICATIONS | WHO IRIS public Discover API | "school food" AND (child* OR adolescen*) | 2015-2025; Publications | 2026-07-10 | 41 | completed |
| WHO_IRIS_Q03_TECHNICAL_DOCUMENTS | WHO IRIS public Discover API | "school food" AND (child* OR adolescen*) | 2015-2025; Technical Documents | 2026-07-10 | 3 | completed |
| WHO_IRIS_Q03_GOVERNING_BODIES_DOCUMENTS | WHO IRIS public Discover API | "school food" AND (child* OR adolescen*) | 2015-2025; Governing Bodies documents | 2026-07-10 | 7 | completed |
| WHO_IRIS_Q04_PUBLICATIONS | WHO IRIS public Discover API | "ending childhood obesity" | 2015-2025; Publications | 2026-07-10 | 47 | completed |
| WHO_IRIS_Q04_TECHNICAL_DOCUMENTS | WHO IRIS public Discover API | "ending childhood obesity" | 2015-2025; Technical Documents | 2026-07-10 | 19 | completed |
| WHO_IRIS_Q04_GOVERNING_BODIES_DOCUMENTS | WHO IRIS public Discover API | "ending childhood obesity" | 2015-2025; Governing Bodies documents | 2026-07-10 | 71 | completed |
| WHO_IRIS_Q05_PUBLICATIONS | WHO IRIS public Discover API | "adolescent nutrition" AND (guideline OR guidance) | 2015-2025; Publications | 2026-07-10 | 14 | completed |
| WHO_IRIS_Q05_TECHNICAL_DOCUMENTS | WHO IRIS public Discover API | "adolescent nutrition" AND (guideline OR guidance) | 2015-2025; Technical Documents | 2026-07-10 | 7 | completed |
| WHO_IRIS_Q05_GOVERNING_BODIES_DOCUMENTS | WHO IRIS public Discover API | "adolescent nutrition" AND (guideline OR guidance) | 2015-2025; Governing Bodies documents | 2026-07-10 | 8 | completed |
| WHO_IRIS_Q06_PUBLICATIONS | WHO IRIS public Discover API | "inappropriate promotion" AND (infant* OR "young children") | 2015-2025; Publications | 2026-07-10 | 27 | completed |
| WHO_IRIS_Q06_TECHNICAL_DOCUMENTS | WHO IRIS public Discover API | "inappropriate promotion" AND (infant* OR "young children") | 2015-2025; Technical Documents | 2026-07-10 | 11 | completed |
| WHO_IRIS_Q06_GOVERNING_BODIES_DOCUMENTS | WHO IRIS public Discover API | "inappropriate promotion" AND (infant* OR "young children") | 2015-2025; Governing Bodies documents | 2026-07-10 | 27 | completed |
| WHO_IRIS_Q07_PUBLICATIONS | WHO IRIS public Discover API | "food labelling" AND (child* OR adolescen*) | 2015-2025; Publications | 2026-07-10 | 57 | completed |
| WHO_IRIS_Q07_TECHNICAL_DOCUMENTS | WHO IRIS public Discover API | "food labelling" AND (child* OR adolescen*) | 2015-2025; Technical Documents | 2026-07-10 | 18 | completed |
| WHO_IRIS_Q07_GOVERNING_BODIES_DOCUMENTS | WHO IRIS public Discover API | "food labelling" AND (child* OR adolescen*) | 2015-2025; Governing Bodies documents | 2026-07-10 | 4 | completed |
| WHO_IRIS_Q08_PUBLICATIONS | WHO IRIS public Discover API | "food systems" AND children AND (guidance OR framework) | 2015-2025; Publications | 2026-07-10 | 173 | completed |
| WHO_IRIS_Q08_TECHNICAL_DOCUMENTS | WHO IRIS public Discover API | "food systems" AND children AND (guidance OR framework) | 2015-2025; Technical Documents | 2026-07-10 | 76 | completed |
| WHO_IRIS_Q08_GOVERNING_BODIES_DOCUMENTS | WHO IRIS public Discover API | "food systems" AND children AND (guidance OR framework) | 2015-2025; Governing Bodies documents | 2026-07-10 | 29 | completed |
| WHO_IRIS_Q09_PUBLICATIONS | WHO IRIS public Discover API | "childhood overweight" AND (policy OR target) | 2015-2025; Publications | 2026-07-10 | 43 | completed |
| WHO_IRIS_Q09_TECHNICAL_DOCUMENTS | WHO IRIS public Discover API | "childhood overweight" AND (policy OR target) | 2015-2025; Technical Documents | 2026-07-10 | 16 | completed |
| WHO_IRIS_Q09_GOVERNING_BODIES_DOCUMENTS | WHO IRIS public Discover API | "childhood overweight" AND (policy OR target) | 2015-2025; Governing Bodies documents | 2026-07-10 | 15 | completed |
| UNICEF_INTERNAL_01 | UNICEF Documents and Reports | child OR children OR adolescent OR infant; food environment OR food marketing OR school food OR food retail; policy OR guidance OR strategy OR regulation OR programme | 2015-2025 | 2026-07-10 | Not estimable | interface blocked security verification |
| UNICEF_WEB_Q01 | UNICEF official domain | site:unicef.org/documents UNICEF children "food environment" guidance policy | 2015-2025 | 2026-07-10 | Not estimable | completed official domain fallback |
| UNICEF_WEB_Q02 | UNICEF official domain | site:unicef.org/documents UNICEF children ("food marketing" OR "food retail") guidance | 2015-2025 | 2026-07-10 | Not estimable | completed official domain fallback |
| UNICEF_WEB_Q03 | UNICEF official domain | site:unicef.org/reports UNICEF child nutrition food systems policy guidance 2015 2025 | 2015-2025 | 2026-07-10 | Not estimable | completed official domain fallback |
| UNICEF_WEB_Q04 | UNICEF official domain | site:unicef.org/media filetype:pdf UNICEF children food environments regulatory guidance | 2015-2025 | 2026-07-10 | Not estimable | completed official domain fallback |
| UNICEF_WEB_Q05 | UNICEF official domain | site:unicef.org/documents/nutrition UNICEF "Programme Guidance" food children global | 2015-2025 | 2026-07-10 | Not estimable | completed official domain fallback |
| UNICEF_WEB_Q06 | UNICEF official domain | site:unicef.org/documents/nutrition UNICEF "Guidance Framework" children food | 2015-2025 | 2026-07-10 | Not estimable | completed official domain fallback |
| UNICEF_WEB_Q07 | UNICEF official domain | site:unicef.org/documents/nutrition UNICEF "Technical Note" "food environments" | 2015-2025 | 2026-07-10 | Not estimable | completed official domain fallback |
| UNICEF_WEB_Q08 | UNICEF official domain | site:unicef.org/documents UNICEF "school food" guidance global | 2015-2025 | 2026-07-10 | Not estimable | completed official domain fallback |
| UNICEF_VERSION_Q01 | UNICEF official domain | site:unicef.org/media "Engaging with the Food and Beverage Industry" "2023" UNICEF PDF | 2015-2025 | 2026-07-10 | Not estimable | completed version recovery |
| UNICEF_INDEX_01 | UNICEF UN Food Systems Summit resource index | Global programme guidance; strategic direction; standards; evidence; and tools listed for children's foods and food environments | 2015-2025 | 2026-07-10 | Not estimable | completed index crosscheck |

# 2 S2. Candidate ledger, versions, and record-specific decisions

The integrated ledger contains 38 unique records: 22 included in the expert-refrozen core corpus, 12 retained for context only, and four excluded. Regional and national documents were excluded by design to keep the administrative level comparable; implementation claims are therefore not generalized to countries, schools, or communities. URLs identify the authoritative landing pages used for version checking.

**Supplementary Table S2A.1. Bibliographic details and stable links, records 1-13 of 38**

| **ID** | **Issuer** | **Year** | **Title** | **Genre** | **Pages** | **URL** |
| --- | --- | --- | --- | --- | --- | --- |
| P001 | WHO | 2023 | Policies to protect children from the harmful impact of food marketing: WHO guideline | guideline | 88 | [Landing page](https://www.who.int/publications/i/item/9789240075412) |
| P002 | WHO | 2016 | Report of the Commission on Ending Childhood Obesity | commission_report | 68 | [Landing page](https://www.who.int/publications/i/item/9789241510066) |
| P003 | UNICEF | 2020 | UNICEF Nutrition Strategy 2020-2030: Nutrition for Every Child | strategy | 110 | [Landing page](https://www.unicef.org/reports/nutrition-strategy-2020-2030) |
| P004 | UNICEF | 2019 | Prevention of Overweight and Obesity in Children and Adolescents: Programming Guidance | programme_guidance | 48 | [Landing page](https://www.unicef.org/documents/prevention-overweight-and-obesity-children-and-adolescents) |
| P005 | UNICEF | 2020 | Prevention of Overweight and Obesity in Children and Adolescents: Advocacy Strategy and Guidance | advocacy_guidance | 44 | [Landing page](https://www.unicef.org/documents/prevention-overweight-and-obesity-children-and-adolescents) |
| P006 | UNICEF | 2022 | Effective regulatory approaches to protect, support and promote better diets and create healthy food environments for children | technical_note | 35 | [Landing page](https://www.unicef.org/documents/effective-regulatory-approaches-food-environments) |
| P007 | UNICEF | 2018 | A Child Rights-Based Approach to Food Marketing | policy_maker_guide | 84 | [Landing page](https://www.unicef.org/documents/child-rights-based-approach-food-marketing) |
| P008 | UNICEF | 2023 | Guidance Framework to Support Healthier Food Retail Environments for Children | guidance_framework | 36 | [Landing page](https://www.unicef.org/documents/nutrition/food-retail-environments) |
| P009 | UNICEF | 2023 | Transforming Food Systems for Children: UNICEF at Work | implementation_overview | 36 | [Landing page](https://www.unicef.org/documents/nutrition/transforming-food-systems-children) |
| P010 | WHO-UNICEF | 2020 | Protecting children from harmful marketing practices | policy_brief | 1 | [Landing page](https://www.who.int/publications/i/item/protecting-children-from-harmful-marketing-practices) |
| P011 | UNICEF | 2024 | Child Food Poverty: Nutrition Deprivation in Early Childhood | flagship_policy_report | 92 | [Landing page](https://www.unicef.org/reports/child-food-poverty) |
| P012 | UNICEF | 2021 | Fed to Fail? The Crisis of Children's Diets in Early Life | flagship_policy_report | 70 | [Landing page](https://www.unicef.org/reports/fed-to-fail-child-nutrition) |
| P013 | UNICEF | 2025 | Feeding Profit: How Food Environments Are Failing Children | flagship_policy_report | 114 | [Landing page](https://www.unicef.org/reports/feeding-profit) |

**Supplementary Table S2A.2. Bibliographic details and stable links, records 14-26 of 38**

| **ID** | **Issuer** | **Year** | **Title** | **Genre** | **Pages** | **URL** |
| --- | --- | --- | --- | --- | --- | --- |
| P014 | UNICEF | 2022 | Shifting the Narrative on Childhood Overweight and Obesity | advocacy_playbook | 24 | [Landing page](https://www.unicef.org/documents/shifting-narrative-childhood-overweight) |
| P015 | WHO-UNICEF | 2026 | The WHO acceleration plan to stop obesity: a joint WHO/UNICEF operational model for designing and implementing the response | operational_model |  | [Landing page](https://www.who.int/publications/i/item/9789240116054) |
| P016 | WHO-UNICEF | 2023 | Taking action to protect children from the harmful impact of food marketing: a child rights-based approach | implementation_toolkit | 76 | [Landing page](https://www.who.int/publications/i/item/9789240047518/) |
| P017 | WHO | 2017 | Guidance on ending the inappropriate promotion of foods for infants and young children: implementation manual | implementation_manual | 51 | [Landing page](https://www.who.int/publications/i/item/9789241513470) |
| P018 | WHO | 2017 | Implementation of the recommendations of the Commission on Ending Childhood Obesity | implementation_plan | 24 | [Landing page](https://iris.who.int/handle/10665/259349) |
| P019 | WHO | 2025 | Global nutrition targets 2030: childhood overweight brief | policy_brief | 11 | [Landing page](https://www.who.int/publications/i/item/B09441) |
| P020 | WHO | 2023 | WHO acceleration plan to stop obesity | advocacy_brochure | 20 | [Landing page](https://www.who.int/publications/i/item/9789240075634) |
| P021 | WHO | 2018 | Guideline: implementing effective actions for improving adolescent nutrition | guideline | 68 | [Landing page](https://www.who.int/publications/i/item/9789241513708) |
| P022 | WHO-UNICEF-UNESCO | 2023 | How school systems can improve health and well-being: topic briefs: nutrition | policy_brief | 8 | [Landing page](https://www.who.int/publications/i/item/9789240064713) |
| P023 | UNICEF | 2020 | Improving Young Children's Diets During the Complementary Feeding Period: UNICEF Programming Guidance | programme_guidance | 76 | [Landing page](https://www.unicef.org/documents/improving-young-childrens-diets-during-complementary-feeding-period-unicef-programming) |
| P024 | UNICEF | 2021 | Nutrition in Middle Childhood and Adolescence: UNICEF Programming Guidance | programme_guidance | 88 | [Landing page](https://www.unicef.org/nutrition/middle-childhood-and-adolescence) |
| P025 | UNICEF-UNSR | 2019 | Protecting Children's Right to a Healthy Food Environment | advocacy_brief | 28 | [Landing page](https://www.unicef.org/documents/protecting-childrens-right-healthy-food-environment) |
| P026 | UNICEF | 2020 | UNICEF Conceptual Framework on Maternal and Child Nutrition | conceptual_framework | 4 | [Landing page](https://www.unicef.org/documents/conceptual-framework-nutrition) |

**Supplementary Table S2A.3. Bibliographic details and stable links, records 27-38 of 38**

| **ID** | **Issuer** | **Year** | **Title** | **Genre** | **Pages** | **URL** |
| --- | --- | --- | --- | --- | --- | --- |
| P027 | UNICEF | 2022 | A Systems Approach to Improving Children's Diets | illustrative_framework | 20 | [Landing page](https://www.unicef.org/documents/systems-approach-improving-childrens-diets) |
| P028 | UNICEF-GAIN | 2018 | The Innocenti Framework on Food Systems for Children and Adolescents | conceptual_framework | 12 | [Landing page](https://www.unicef.org/documents/innocenti-framework-food-systems-children-and-adolescents) |
| P029 | UNICEF | 2023 | The global climate crisis is a child nutrition crisis: UNICEF Agenda for Child Nutrition and Climate Action | agenda | 6 | [Landing page](https://www.unicef.org/documents/global-climate-crisis-child-nutrition-crisis) |
| P030 | UNICEF | 2021 | Food-Based Dietary Guidelines | programme_guidance | 160 | [Landing page](https://www.unicef.org/documents/food-based-dietary-guidelines) |
| P031 | UNICEF | 2022 | Engaging Business for Maternal and Child Nutrition: UNICEF Programming Guidance | programme_guidance | 55 | [Landing page](https://www.unicef.org/documents/guidance-engaging-business-nutrition) |
| P032 | UNICEF | 2024 | Building synergies between child nutrition and social protection to address malnutrition and poverty | programme_guidance | 48 | [Landing page](https://www.unicef.org/reports/building-synergies-between-child-nutrition-and-social-protection-address-malnutrition) |
| P033 | WHO | 2026 | Policies and interventions to create healthy school food environments: WHO guideline | guideline |  | [Landing page](https://www.who.int/publications/i/item/9789240118324) |
| P034 | WHO-UNICEF | 2022 | Protecting children from the harmful impact of food marketing: policy brief | policy_brief | 14 | [Landing page](https://iris.who.int/handle/10665/354606) |
| P035 | WHO | 2023 | Guidance on regulatory measures aimed at restricting digital marketing of breast-milk substitutes | regulatory_guidance | 24 | [Landing page](https://iris.who.int/handle/10665/374182) |
| P036 | WHO | 2024 | Sponsorship of health professional associations by manufacturers and distributors of commercial milk formula: model policy | model_policy | 4 | [Landing page](https://iris.who.int/handle/10665/378985) |
| P037 | UNICEF | 2023 | Engaging with the Food and Beverage Industry: UNICEF Programme Guidance | programme_guidance | 19 | [Landing page](https://www.unicef.org/documents/nutrition/engaging-food-and-beverage-industry) |
| P038 | UNICEF | 2026 | Engaging with the Food and Beverage Industry: UNICEF Guidance for Programmes and Partnerships | programme_and_partnership_guidance |  | [Landing page](https://www.unicef.org/documents/nutrition/engaging-food-and-beverage-industry) |

**Supplementary Table S2B. Version relationships and record-specific decisions for all 38 candidates**

| **ID** | **Decision** | **Version** | **Record-specific rationale** |
| --- | --- | --- | --- |
| P001 | include core | Authoritative full version | Child food-marketing policy is the document's explicit subject. |
| P002 | include core | Authoritative full version | Formal commission recommendations contain substantial food-environment policy action. |
| P003 | include core | Edition 2021 | Global child-nutrition strategy gives food systems and food environments a central policy role. |
| P004 | include core | Authoritative full version | Child-overweight programming guidance contains substantial food-environment and regulatory action. |
| P005 | include core | Authoritative full version | UNICEF global-HQ, 2020. An advocacy strategy telling country offices how to press governments on food-environment regulation, monitoring and enforcement. It urges action rather than issuing recommendations, so it sits a step back from the normative instruments; on the other criteria it is a clear fit, right issuer, in window, on the food-environment domain. I kept it core, near the boundary. |
| P006 | include core | Authoritative full version | Government-led food-environment regulation is the central function. |
| P007 | include core | Authoritative full version | Government and stakeholder recommendations on child food marketing are explicit. |
| P008 | include core | Authoritative full version | Food-retail environments and operational responsibilities are the direct focus. |
| P009 | context only | Authoritative full version | Implementation examples and organizational positioning dominate over normative guidance. |
| P010 | context only | Authoritative full version | One-page policy brief cannot contribute stable chunks to the main model. |
| P011 | context only | Authoritative full version | Agenda-setting report is analytically valuable but not genre-comparable with normative guidance. |
| P012 | context only | Authoritative full version | Flagship evidence report is retained outside the normative core. |
| P013 | context only | Authoritative full version | Flagship report is highly relevant but would dominate the corpus by length and genre. |
| P014 | context only | Authoritative full version | Criteria: I05; I06; E07 (Tier B contextual genre) Evidence: PDF pp. 4, 9-10: overview and narrative-framing recommendations; document explicitly identifies itself as an advocacy playbook. Retrospective joint rationale: Shifting the Narrative on Childhood Overweight and Obesity The advocacy playbook is retained for framing context but excluded from the primary corpus to avoid a unique UNICEF communication-genre effect. |
| P015 | exclude | Authoritative full version | Official publication date falls after 31 December 2025. |
| P016 | include core | Authoritative full version | Joint WHO-UNICEF government-facing implementation toolkit directly meets all criteria. |
| P017 | include core | Authoritative full version | Implementation manual regulates promotion of foods for infants and young children. |
| P018 | include core | Authoritative full version | Global implementation plan translates ECHO recommendations into policy action. |
| P019 | include core | Authoritative full version | Brief directly specifies interventions and policies for the childhood-overweight target. |
| P020 | exclude | Authoritative full version | Life-course obesity brochure has limited substantive child-specific food-environment coverage. |
| P021 | include core | Authoritative full version | Clear fit. WHO-HQ 2018 guideline on adolescent nutrition, in window, in English, operational recommendations, several directly on food environments. No reservations. |
| P022 | context only | Authoritative full version | Short school-nutrition topic brief is retained for context rather than topic modelling. |
| P023 | include core | Authoritative full version | Young-child programming guidance gives the food system a substantial action framework. |
| P024 | include core | Authoritative full version | School and wider food environments are a substantial programme priority. |
| P025 | include core | Authoritative full version | Criteria: I05; I06; I07 Evidence: PDF pp. 17-21: government duties, regulation, marketing restrictions, affordability, and direct action recommendations under a child-rights framework. Retrospective joint rationale: Protecting Children's Right to a Healthy Food Environment The brief performs a substantive rights-based policy function and contains direct recommendations for government and food-system action. |
| P026 | context only | Authoritative full version | Conceptual diagram provides background but insufficient policy text. |
| P027 | context only | Authoritative full version | Lived-experience examples support interpretation but are not normative guidance. |
| P028 | context only | Authoritative full version | Conceptual food-systems framework is background evidence rather than implementation guidance. |
| P029 | context only | Authoritative full version | Short climate agenda contains food-environment action but is adjacent in domain and genre. |
| P030 | context only | Authoritative full version | The file is a review of national dietary guidelines rather than UNICEF policy guidance. |
| P031 | include core | Authoritative full version | Criteria: I05; I06; I07 Evidence: PDF pp. 15, 21, 23, 29-33: responsible business practice, exclusion criteria, programme-cycle integration, due diligence, monitoring, and safeguards. Retrospective joint rationale: Engaging Business for Maternal and Child Nutrition: UNICEF Programming Guidance The programme guidance contains substantive child-nutrition business-governance safeguards and implementation guidance despite mixed maternal content. |
| P032 | include core | Authoritative full version | Criteria: I05; I06; I07 Evidence: PDF pp. 12, 18, 25, 33: child-nutrition/social-protection framework, policy mapping, social transfers, affordability, and implementation actions. Retrospective joint rationale: Building synergies between child nutrition and social protection to address malnutrition and poverty Food affordability and child-sensitive social-protection action are central enough to support policy and implementation analysis. |
| P033 | exclude | Authoritative full version | Official item page confirms publication on 27 January 2026. |
| P034 | include core | Authoritative full version | Global policy brief gives government-facing and comprehensive child food-marketing policy options. |
| P035 | include core | Authoritative full version | Global guidance gives detailed Member-State regulatory recommendations for digital marketing. |
| P036 | include core | Authoritative full version | WHO-HQ, 2024, four pages on IRIS. Draft model-policy text for restricting commercial milk formula manufacturers' sponsorship of health professional associations. It's short, but it does something the other documents don't: it gives governments ready-to-adopt legislative language. That concreteness is what makes it core. |
| P037 | include core | Authoritative full version | Global UNICEF guidance makes public policy and child food-environment governance central to ten minimum engagement parameters. |
| P038 | exclude | 2026 replacement; outside window | Current landing-page replacement was published in 2026 and is outside the frozen period. |

# 3 S3. Provisional cluster evidence and document-level prevalence

The primary BERTopic output is reported as six provisional clusters in the expert-refrozen corpus. Cluster labels are descriptors, not independently established categories. Cluster 3 and Cluster 5 descriptors were revised in response to peer review; the historical consensus labels remain preserved in the audit files, with the crosswalk recorded in revision_topic_label_crosswalk.csv. The 18 evidence chunks preserve the pre-amendment masked label-review mapping shown to the expert. Most displays use a deterministic 80-word window maximizing overlap with the cluster's top-15 terms. P006 and P031 use fixed sentence-boundary contexts from the same frozen chunks because the original windows contained OCR-fragmented or contents-page text; the original windows, hashes, and exception rationales remain in revision_expert_masked_evidence.csv. These procedures improve context and auditability but do not eliminate selection bias. The expert accepted all six descriptors and changed three boundary decisions. After those changes were applied, the complete 22-document refit retained six clusters with an identity lexical mapping; same-cluster top-10-term Jaccard similarity ranged from 0.667 to 1.000. The 0.60 transfer gate was a revision-stage operational rule, not a preregistered threshold; the completed independent review accepted all six descriptors.

## 3.1 Cluster 0: Food-marketing restrictions and children's rights in food environments

Top 15 weighted terms: marketing; food; food marketing; rights; children; childrens; unhealthy; impact; health; policies; policy; healthy; evidence; foods; child. Document coverage: 9 documents. Dominant source: P016 (27.4% of assigned chunks). Reporting status: retain.

Revision rationale: Original label remains congruent with weighted terms and evidence excerpts

**Supplementary Table S3.1. Masked contextual evidence from three distinct documents**

| **Record/source rank** | **Locator** | **Source and membership** | **Audited contextual excerpt** |
| --- | --- | --- | --- |
| P001 / rank 2 | P001_C0011; p. 12; A wide variety of marketing strategies that were likely to appeal to children were reported by studies, and | WHO; 2023; membership 1.000 | “... television advertising of HFSS foods, and reduced food and beverage advertising revenue on children’s channels; these changes were considered favourable to public health. The overall evidence on the effect of policies on children’s exposure to food marketing and the power of food marketing was inconsistent. Analyses by policy design elements, however, showed that reductions in children’s exposure to food marketing were more often found with:  mandatory policies;  policies designed to restrict food marketing to children, including those older ...” |
| P006 / rank 5 | P006_C0004; p. 5-6; The [ORGANIZATION] Guidance on the Prevention of Overweight | UNICEF; 2022; membership 1.000 | “Thereafter, [ORGANIZATION] offices are encouraged to scale up country-level support, together with governments and counterparts, to improve the enabling environment. This includes the development and implementation of regulatory actions as well as monitoring and enforcement measures. The [ORGANIZATION] Guidance recommends the following FIVE mutually supportive regulatory actions:” |
| P007 / rank 6 | P007_C0014; p. 11-13; Part 2 discusses the extent to which children are exposed to unhealthy food marketing, and | UNICEF; 2018; membership 1.000 | “... It then examines the evidence on how marketing influences children’s food preferences, purchase requests and consumption patterns. New marketing channels and ever-evolving techniques, combined with escalating exposure, have a powerful effect on children. In evidence gathered over the past 20 years, unhealthy food marketing has been identified as a factor that contributes to growing rates of overweight and obesity. In May 2010, the World Health Assembly unanimously adopted the [ORGANIZATION] Recommendations, marking the issue of unhealthy food marketing to children ...” |

## 3.2 Cluster 1: Childhood overweight and obesity: epidemiological burden and global trends

Top 15 weighted terms: obesity; overweight; children; childhood; years; childhood obesity; prevalence; health; countries; overweight obesity; adolescents; global; growth; world; iron. Document coverage: 18 documents. Dominant source: P005 (18.7% of assigned chunks). Reporting status: retain.

Revision rationale: Descriptor is explicitly treated as a burden-description cluster rather than a policy instrument cluster

**Supplementary Table S3.2. Masked contextual evidence from three distinct documents**

| **Record/source rank** | **Locator** | **Source and membership** | **Audited contextual excerpt** |
| --- | --- | --- | --- |
| P002 / rank 1 | P002_C0012; p. 18; INTRODUCTION | WHO; 2016; membership 1.000 | “... contributed to the increased longevity observed in the world. In 2014, an estimated 41 million children under 5 years of age were affected by overweight or obesity (1) (defined as the proportion of children with weight-for-height z-score values more than 2 SDs and more than 3 SDs, respectively, from the [ORGANIZATION] growth standard median (2)). Figure 1 shows the prevalence of overweight children under 5 years of age worldwide. In Africa, the number of children [ORGANIZATION] are overweight or obese ...” |
| P003 / rank 3 | P003_C0018; p. 15; [ORGANIZATION]/World Bank Group Joint Child Malnutrition | UNICEF; 2020; membership 1.000 | “... • 144 million children under 5 – about 21 per cent – are stunted. In South Asia and sub-Saharan Africa, stunting affects one third of children under 5. About half (54 per cent) of all children [ORGANIZATION] are stunted live in Asia and more than one third (40 per cent) live in Africa. Ninety-one per cent of the children [ORGANIZATION] are stunted globally live in low-income and lower-middle-income countries (27 and 64 per cent respectively). • 47 million children under ...” |
| P004 / rank 5 | P004_C0010; p. 13; 19 years of age are collected in multiple surveys, | UNICEF; 2019; membership 1.000 | “... higher income countries, predominantly an urban and middle-class phenomenon of higher middle-income countries. In established overweight epidemics, the burden of overweight has affected lower socioeconomic groups disproportionally. New evidence suggests that these patterns are changing, with increasing prevalence of overweight among both urban and rural poor children and children in low-and-middle-income countries. 9 Middle income countries account for over three quarters of all children with overweight: 38 per cent of all children with overweight live in lower middleincome countries while ...” |

## 3.3 Cluster 2: Nutrition-sensitive social protection and service delivery

Top 15 weighted terms: nutrition; social; social protection; protection; programmes; services; child; children; support; systems; national; diets; transfers; child nutrition; water. Document coverage: 5 documents. Dominant source: P032 (39.1% of assigned chunks). Reporting status: retain with caution.

Revision rationale: Limited five-document coverage remains explicitly caution-qualified after the expert-refrozen refit

**Supplementary Table S3.3. Masked contextual evidence from three distinct documents**

| **Record/source rank** | **Locator** | **Source and membership** | **Audited contextual excerpt** |
| --- | --- | --- | --- |
| P032 / rank 1 | P032_C0017; p. 15; MONITORING AND LEARNING IMPLEMENTATION AND DELIVERY | UNICEF; 2024; membership 1.000 | “... nutrition services. Action 7: Link to wider systems efforts for sustained food, nutrition and income security. Action 8: Make child nutrition and social protection programmes shock-responsive to mitigate increased poverty, vulnerability and prevent malnutrition. Action 9: Strengthen local implementation capacities, including nutrition and social protection workforces and information systems. Action 10: Monitor, track, understand impact and learn, including for the purposes of scale-up. This section describes 10 actions that can be taken to strengthen synergies between nutrition and social protection ...” |
| P023 / rank 4 | P023_C0037; p. 28; Not recorded | UNICEF; 2020; membership 0.925 | “... child malnutrition by narrowing the gaps in economic access and promoting women’s decisionmaking. Certain types of social protection programmes, such as cash transfers, can increase the household budget for food purchase, which in turn influences what children eat.156 In humanitarian situations, cash transfers can prevent negative coping responses to food scarcity (such as reducing the number of meals per day), improve dietary intake and access to diverse foods for children.157 The integration of nutrition counselling and SBCC within social protection ...” |
| P003 / rank 6 | P003_C0102; p. 75; Not recorded | UNICEF; 2020; membership 0.866 | “... workers. 5. Synergistic community-based programmes for nutrition: [ORGANIZATION] nutrition programmes strengthen partnerships with water, hygiene and sanitation programmes to improve outreach and maximize the nutrition impact of community-based initiatives (e.g., community-led total sanitation, community-based nutrition counselling, or the community management of wasting). This includes generating evidence on the impact of such approaches on nutrition outcomes for children. Safe and palatable drinking water is an essential component of good diets, while safe sanitation and hygiene services protect children from nutrient losses.” |

## 3.4 Cluster 3: School, physical-activity, and healthy food environments

Top 15 weighted terms: physical; physical activity; activity; foods; healthy; children; school; beverages; nutrition; food; health; education; appropriate; schools; including. Document coverage: 10 documents. Dominant source: P002 (22.6% of assigned chunks). Reporting status: retain.

Revision rationale: Revised descriptor makes the leading physical-activity and school terms visible and avoids implying that nutrition standards alone define the cluster

**Supplementary Table S3.4. Masked contextual evidence from three distinct documents**

| **Record/source rank** | **Locator** | **Source and membership** | **Audited contextual excerpt** |
| --- | --- | --- | --- |
| P002 / rank 1 | P002_C0008; p. 13-14; ACTIONS AND RESPONSIBILITIES FOR: | WHO; 2016; membership 1.000 | “Establish standards for meals provided in schools, or foods and beverages sold in schools, that meet healthy nutrition guidelines. Eliminate the provision or sale of unhealthy foods, such as sugar-sweetened beverages and energy-dense, nutrient-poor foods, in the school environment. Ensure access to potable water in schools and sports facilities. Require inclusion of nutrition and health education within the core curriculum of schools. Improve the nutrition literacy and skills of parents and caregivers. Make food preparation classes available to children, their ...” |
| P004 / rank 4 | P004_C0036; p. 32; Young Children;96 | UNICEF; 2019; membership 1.000 | “... the Guidance on Ending the • implementation of the set of recommendations on the marketing of foods and non-alcoholic beverages to children;97 • adoption of health-related food taxes, such as increased taxes on sugary and sweet beverages and ‘junk food’ and subsidies for healthy foods;98,99,100,101,102 • reformulation of processed foods including • adoption of front-of-pack nutrition labelling requirements that identify foods that are high in salt, sugar and fats103,104; and • use of urban planning regulations to promote healthy food ...” |
| P018 / rank 6 | P018_C0016; p. 16-17; Not recorded | WHO; 2017; membership 1.000 | “... Set standards for physical activity in child-care settings. • Provide guidance to carers on the provision of safe and developmentally-appropriate physical activity, active play and active recreation for all children. Provide guidance on appropriate sleep time, sedentary or screen-time, and physical activity or active play for the 2–5 years of age group. • Develop guidance on physical activity for children under 5 years of age, including age-appropriate activities and ideas to support and encourage participation in physical activity at home ...” |

## 3.5 Cluster 4: Infant and young-child food-promotion controls

Top 15 weighted terms: digital; infants; young; marketing; promotion; code; infants young; health; young children; products; breastmilk; foods; measures; foods infants; including. Document coverage: 8 documents. Dominant source: P017 (48.2% of assigned chunks). Reporting status: retain with caution.

Revision rationale: Single-document concentration remains explicitly caution-qualified

**Supplementary Table S3.5. Masked contextual evidence from three distinct documents**

| **Record/source rank** | **Locator** | **Source and membership** | **Audited contextual excerpt** |
| --- | --- | --- | --- |
| P006 / rank 2 | P006_C0028; p. 16; the Office of the United Nations High Commissioner | UNICEF; 2022; membership 1.000 | “... media and digital marketing, to promote BMS, bottles, and teats to the general public and promotion through health care systems; free samples to mothers; using sales inducements such as special offers or price reductions; publicizing health or nutrition claims on labels or other BMS materials; idealizing BMS products in text or images; providing free supplies of BMS, bottles, or teats to health facilities; and sponsoring the education and meetings of health workers. It is thus important that measures cover all ...” |
| P017 / rank 4 | P017_C0034; p. 24; Not recorded | WHO; 2017; membership 1.000 | “... Follow-up formula should, therefore, not be promoted. Growing-up milk (also known as growing-up formula, toddler milk or formulated milk) This product is targeted at infants and young children from 1 year old (sometimes younger) to 3 years old. Often, the product name is similar to a company’s formula products, with a figure “3” added on. Where growing-up milks are marketed as suitable for feeding young children up to the age of 36 months, they fall under the International Code definition ...” |
| P035 / rank 6 | P035_C0014; p. 16-17; WHA61.20, WHA63.23 and WHA69.9) in | WHO; 2023; membership 1.000 | “... and caregivers of infants and young children in the digital environment. measures should prohibit the sponsorship of online meetings of health professionals and scientific meetings, including webinars, e-learning courses and information dissemination through online scientific communications, including advertising in digital medical journals and on e-learning platforms by manufacturers and distributors of products within the scope of the Code or foods for infants and young children (10,11). Regulatory measures should prohibit the promotion of products within the scope of the Code, ...” |

## 3.6 Cluster 5: Business engagement and food-industry governance

Top 15 weighted terms: business; industry; engagement; upf; nutrition; programme; food; unicefs; companies; engaging; children; practices; programming; childrens; outcomes. Document coverage: 5 documents. Dominant source: P031 (78.0% of assigned chunks). Reporting status: exclude from comparative interpretation.

Revision rationale: Revised descriptor reflects engagement, safeguards, due diligence, and source concentration without implying demonstrated child outcomes

**Supplementary Table S3.6. Masked contextual evidence from three distinct documents**

| **Record/source rank** | **Locator** | **Source and membership** | **Audited contextual excerpt** |
| --- | --- | --- | --- |
| P003 / rank 1 | P003_C0117; p. 86; Not recorded | UNICEF; 2020; membership 1.000 | “... to adhere to the United Nations Guiding Principles on Business and Human Rights,89 the Child Rights and Business Principles,90 and to the Convention on the Rights of the Child’s provision to uphold children’s right to nutrition. Five key entry points for engaging with business to improve maternal and child nutrition include: • Business as a provider of essential goods and services for children and families, for example, through industry support for large-scale food fortification and fortified complementary foods; • Business ...” |
| P031 / rank 2 | P031_C0002; p. 4-6; Figure 1: [ORGANIZATION] Nutrition Strategy 2020–2030: Nutrition, For Every Child 7 | UNICEF; 2022; membership 1.000 | “identifies the potential of engaging business when the private sector is uniquely positioned to help [ORGANIZATION] achieve priority nutrition outcomes, at the same time as being aware of risks that need to be managed. It intends to provide [ORGANIZATION] teams with practical and evidence-based guidance on effectively engaging the private sector to implement the Nutrition Strategy. The content of this Programming Guidance is based on the lessons and insights generated by a review of [ORGANIZATION]’s past and recent experiences in engaging business for nutrition outcomes across several regions. Ten case studies were developed from this review and are referenced in the Programming Guidance to provide practical country and programme examples.” |
| P037 / rank 5 | P037_C0005; p. 8; Not recorded | UNICEF; 2023; membership 1.000 | “[ORGANIZATION] recognizes the positive impact that businesses can have on children’s rights. Therefore, [ORGANIZATION] programmes seek to mobilize private sector’s resources, expertise, and voice, and advocate for private sector practices, products and services that are aligned with children’s rights. This is the case, for example, of [ORGANIZATION]’s engagement with the food industry on large-scale food fortification of staple cereals and condiments, including wheat flour fortification with essential micronutrients and universal salt iodization. However, when core business practices and products are ...” |

**Supplementary Table S4. Document-level cluster shares (% of all primary chunks; outliers retained in the denominator)**

| **Record** | **Issuer** | **Period** | **Chunks** | **C0** | **C1** | **C2** | **C3** | **C4** | **C5** |
| --- | --- | --- | --- | --- | --- | --- | --- | --- | --- |
| P001 | WHO | 2020-2025 | 91 | 90.1 | 1.1 | 0.0 | 0.0 | 0.0 | 0.0 |
| P002 | WHO | 2015-2019 | 48 | 2.1 | 43.8 | 0.0 | 39.6 | 0.0 | 0.0 |
| P003 | UNICEF | 2020-2025 | 137 | 0.0 | 22.6 | 46.7 | 0.0 | 0.0 | 3.6 |
| P004 | UNICEF | 2015-2019 | 42 | 0.0 | 23.8 | 0.0 | 28.6 | 0.0 | 0.0 |
| P005 | UNICEF | 2020-2025 | 55 | 0.0 | 61.8 | 0.0 | 9.1 | 0.0 | 1.8 |
| P006 | UNICEF | 2020-2025 | 56 | 16.1 | 3.6 | 0.0 | 12.5 | 16.1 | 0.0 |
| P007 | UNICEF | 2015-2019 | 90 | 76.7 | 6.7 | 0.0 | 1.1 | 0.0 | 0.0 |
| P008 | UNICEF | 2020-2025 | 48 | 85.4 | 2.1 | 0.0 | 0.0 | 6.2 | 0.0 |
| P016 | WHO-UNICEF | 2020-2025 | 142 | 64.1 | 0.7 | 0.0 | 0.0 | 0.7 | 0.0 |
| P017 | WHO | 2015-2019 | 54 | 0.0 | 0.0 | 0.0 | 0.0 | 74.1 | 0.0 |
| P018 | WHO | 2015-2019 | 18 | 0.0 | 38.9 | 0.0 | 50.0 | 0.0 | 0.0 |
| P019 | WHO | 2020-2025 | 16 | 0.0 | 43.8 | 0.0 | 37.5 | 0.0 | 0.0 |
| P021 | WHO | 2015-2019 | 74 | 0.0 | 39.2 | 0.0 | 12.2 | 0.0 | 0.0 |
| P023 | UNICEF | 2020-2025 | 67 | 0.0 | 14.9 | 56.7 | 0.0 | 10.4 | 0.0 |
| P024 | UNICEF | 2020-2025 | 53 | 1.9 | 22.6 | 11.3 | 28.3 | 0.0 | 1.9 |
| P025 | joint_other | 2015-2019 | 36 | 58.3 | 16.7 | 0.0 | 2.8 | 0.0 | 0.0 |
| P031 | UNICEF | 2020-2025 | 100 | 0.0 | 2.0 | 1.0 | 0.0 | 2.0 | 92.0 |
| P032 | UNICEF | 2020-2025 | 72 | 0.0 | 1.4 | 97.2 | 0.0 | 0.0 | 0.0 |
| P034 | WHO-UNICEF | 2020-2025 | 19 | 89.5 | 5.3 | 0.0 | 0.0 | 0.0 | 0.0 |
| P035 | WHO | 2020-2025 | 16 | 0.0 | 0.0 | 0.0 | 0.0 | 100.0 | 0.0 |
| P036 | WHO | 2020-2025 | 5 | 0.0 | 0.0 | 0.0 | 0.0 | 100.0 | 0.0 |
| P037 | UNICEF | 2020-2025 | 20 | 0.0 | 0.0 | 0.0 | 0.0 | 0.0 | 95.0 |

# 4 S4. Human-coding protocol, reliability, and adjudication

Two authors independently coded a deliberately topic-balanced 84-chunk validation sample selected and locked under the pre-expert 19-document freeze. It was not extended after three documents were added. The sample had no corpus-level inclusion probabilities, so code frequencies are not estimates of instrument prevalence, policy priority, or implementation readiness. The coding outputs are retained solely to audit reliability, codebook behavior, and adjudication; they do not support the substantive portfolio argument. No numerical reliability threshold was preregistered. The reporting gate was rule-based: dimensions with substantial disagreement required codebook clarification, masked recoding, and adjudication before interpretive use. Because round-2 recoding used the same validation sample, its agreement estimates post-calibration reproducibility within that sample rather than holdout validation.

**Supplementary Table S5. Complete primary codebook**

| **Dimension** | **Code** | **Label** | **Operational definition** |
| --- | --- | --- | --- |
| problem_frame | F_IND | Individual behaviour | Individual knowledge, preference, choice, self-control, or behaviour is the primary frame. |
| problem_frame | F_FAM | Family and care | Caregiving, household feeding, parental practice, or family resources are primary. |
| problem_frame | F_INST | Institutional setting | School, health, childcare, retail, or another institutional environment is primary. |
| problem_frame | F_MARKET | Market and commercial | Marketing, pricing, retail, product formulation, availability, or industry practice is primary. |
| problem_frame | F_STRUCT | Structural conditions | Poverty, inequity, social protection, conflict, climate, or food-system structure is primary. |
| problem_frame | F_RIGHTS | Rights and equity | Child rights, equity, non-discrimination, or duty-bearer obligations provide the primary frame. |
| problem_frame | F_NONE | No clear frame | No frame is sufficiently explicit. |
| problem_frame | F_MIXED | Mixed | Two or more frames are equally central and cannot be prioritized. |
| policy_instrument | I_REG | Regulation | Binding law, standard, restriction, enforcement, or compliance is primary. |
| policy_instrument | I_FISC | Fiscal measure | Tax, subsidy, procurement, budget, transfer, or financial incentive is primary. |
| policy_instrument | I_INFO | Information and education | Labelling, education, counselling, communication, or public information is primary. |
| policy_instrument | I_SERVICE | Service or programme | School meals, health/nutrition service, social protection, or programme delivery is primary. |
| policy_instrument | I_PARTNER | Partnership or voluntary action | Collaboration, voluntary commitment, partnership, or self-regulation is primary. |
| policy_instrument | I_ACCOUNT | Monitoring and accountability | Target, indicator, reporting, surveillance, evaluation, or review is primary. |
| policy_instrument | I_NONE | No clear instrument | No policy instrument is sufficiently explicit. |
| policy_instrument | I_MIXED | Mixed | Two or more instruments are equally central and cannot be prioritized. |
| implementation_specificity | S0 | Principle only | Normative aspiration without a concrete action. |
| implementation_specificity | S1 | Explicit action | A concrete recommendation is stated, but responsibility is not assigned. |
| implementation_specificity | S2 | Action plus owner | A concrete action and responsible actor are both explicit. |
| implementation_specificity | S3 | Operational mechanism | Action and owner are supplemented by a target, timeline, finance, enforcement, monitoring, or delivery mechanism. |

Revision rules between coding rounds. Problem framing was recoded for all 84 units after clarifying the distinction among individual, family, institutional, market, rights, and structural frames. Implementation specificity was recoded for 66 non-S0 units after clarifying S1 versus S2; the 18 round-1 S0 agreements remained locked. Policy instruments were carried forward because round-1 agreement was high; its 11 disagreements were adjudicated. Adjudicators could retain Coder A, retain Coder B, or assign a third code only when the recorded rationale identified the controlling text rule.

**Supplementary Table S6.1. Round 1 agreement metrics**

| **Dimension** | **Scope** | **n** | **Exact** | **Kappa** | **AC1** | **Decision gate** |
| --- | --- | --- | --- | --- | --- | --- |
| problem_frame | all 84 | 84 | 0.464 | 0.317 | 0.401 | calibrate codebook and recode all 84 |
| policy_instrument | all 84 | 84 | 0.869 | 0.839 | 0.852 | retain round1 no recode |
| implementation_specificity | all 84 | 84 | 0.690 | 0.551 | 0.606 | clarify S1 S2 and recode 66 non S0 units |

**Supplementary Table S6.2. Round 2 agreement metrics**

| **Dimension** | **Scope** | **n** | **Exact** | **Kappa** | **AC1** | **Decision gate** |
| --- | --- | --- | --- | --- | --- | --- |
| problem_frame | all_84 | 84 | 0.488 | 0.389 | 0.423 | recommend exclusion pending author approval |
| policy_instrument | all_84 | 84 | 0.869 | 0.839 | 0.852 | adjudicate 11 round1 disagreements |
| implementation_specificity | all_84_including_18_locked_S0 | 84 | 0.964 | 0.946 | 0.954 | pass reliability adjudicate 3 disagreements |
| implementation_specificity | 66_recoded_units_only | 66 | 0.955 | 0.930 | 0.942 | sensitivity only |

**Supplementary Table S7.1. Complete Round 1 problem frame confusion matrix**

| **Coder A \ Coder B** | **F_FAM** | **F_IND** | **F_INST** | **F_MARKET** | **F_MIXED** | **F_NONE** | **F_RIGHTS** | **F_STRUCT** |
| --- | --- | --- | --- | --- | --- | --- | --- | --- |
| F_FAM | 2 | 0 | 0 | 0 | 0 | 0 | 0 | 2 |
| F_IND | 1 | 0 | 0 | 0 | 0 | 0 | 0 | 6 |
| F_INST | 0 | 0 | 6 | 0 | 0 | 0 | 2 | 6 |
| F_MARKET | 0 | 0 | 0 | 3 | 0 | 0 | 11 | 11 |
| F_MIXED | 0 | 0 | 0 | 0 | 0 | 0 | 0 | 0 |
| F_NONE | 0 | 0 | 0 | 0 | 0 | 0 | 0 | 0 |
| F_RIGHTS | 0 | 0 | 0 | 0 | 0 | 0 | 9 | 1 |
| F_STRUCT | 0 | 0 | 0 | 0 | 0 | 0 | 5 | 19 |

**Supplementary Table S7.2. Complete Round 1 policy instrument confusion matrix**

| **Coder A \ Coder B** | **I_ACCOUNT** | **I_FISC** | **I_INFO** | **I_MIXED** | **I_NONE** | **I_PARTNER** | **I_REG** | **I_SERVICE** |
| --- | --- | --- | --- | --- | --- | --- | --- | --- |
| I_ACCOUNT | 6 | 0 | 0 | 0 | 0 | 0 | 0 | 0 |
| I_FISC | 0 | 2 | 0 | 0 | 0 | 0 | 0 | 0 |
| I_INFO | 1 | 0 | 5 | 0 | 0 | 1 | 0 | 0 |
| I_MIXED | 0 | 0 | 0 | 0 | 0 | 0 | 0 | 0 |
| I_NONE | 0 | 0 | 0 | 0 | 17 | 0 | 0 | 0 |
| I_PARTNER | 1 | 0 | 0 | 0 | 0 | 5 | 0 | 0 |
| I_REG | 2 | 2 | 1 | 1 | 0 | 0 | 19 | 0 |
| I_SERVICE | 2 | 0 | 0 | 0 | 0 | 0 | 0 | 19 |

**Supplementary Table S7.3. Complete Round 1 implementation specificity confusion matrix**

| **Coder A \ Coder B** | **S0** | **S1** | **S2** | **S3** |
| --- | --- | --- | --- | --- |
| S0 | 18 | 0 | 0 | 0 |
| S1 | 0 | 24 | 26 | 0 |
| S2 | 0 | 0 | 16 | 0 |
| S3 | 0 | 0 | 0 | 0 |

**Supplementary Table S7.4. Complete Round 2 problem frame confusion matrix**

| **Coder A \ Coder B** | **F_FAM** | **F_IND** | **F_INST** | **F_MARKET** | **F_MIXED** | **F_NONE** | **F_RIGHTS** | **F_STRUCT** |
| --- | --- | --- | --- | --- | --- | --- | --- | --- |
| F_FAM | 2 | 0 | 1 | 0 | 0 | 0 | 0 | 3 |
| F_IND | 0 | 0 | 0 | 0 | 0 | 0 | 0 | 6 |
| F_INST | 0 | 0 | 8 | 0 | 0 | 0 | 4 | 10 |
| F_MARKET | 0 | 0 | 2 | 6 | 0 | 0 | 8 | 9 |
| F_MIXED | 0 | 0 | 0 | 0 | 0 | 0 | 0 | 0 |
| F_NONE | 0 | 0 | 0 | 0 | 0 | 1 | 0 | 0 |
| F_RIGHTS | 0 | 0 | 0 | 0 | 0 | 0 | 14 | 0 |
| F_STRUCT | 0 | 0 | 0 | 0 | 0 | 0 | 0 | 10 |

**Supplementary Table S7.5. Complete Round 2 implementation specificity confusion matrix**

| **Coder A \ Coder B** | **S0** | **S1** | **S2** | **S3** |
| --- | --- | --- | --- | --- |
| S0 | 34 | 0 | 0 | 0 |
| S1 | 0 | 27 | 3 | 0 |
| S2 | 0 | 0 | 20 | 0 |
| S3 | 0 | 0 | 0 | 0 |

**Supplementary Table S8. Complete consensus-adjudication log for retained dimensions**

| **Dimension** | **Sample** | **A** | **B** | **Consensus** | **Relationship** | **Recorded reason** |
| --- | --- | --- | --- | --- | --- | --- |
| policy_instrument | V002 | I_REG | I_INFO | I_REG | coder_a | Text prescribes mandatory marketing-content restrictions ("Messages must include", "No images or text") for infant foods; binding prohibitions are primary, not mere information provision. |
| policy_instrument | V008 | I_REG | I_FISC | I_REG | coder_a | Text frames the problem as absence of adequate regulatory frameworks and industry resistance to evidence-based policies and regulation; fiscal details describe industry profits, not a fiscal policy instrument. |
| policy_instrument | V022 | I_INFO | I_ACCOUNT | I_FISC | third_code | Substantive content details fiscal measures (taxes on unhealthy foods, subsidies on fruits/vegetables, SSB taxes) as the primary policy instrument; guidance-resource listing is secondary framing. |
| policy_instrument | V028 | I_INFO | I_PARTNER | I_PARTNER | coder_b | Text describes voluntary retailer-led digital initiatives (apps, loyalty programmes) as the primary instrument; information features are embedded within voluntary partnership actions by food retailers. |
| policy_instrument | V030 | I_SERVICE | I_ACCOUNT | I_SERVICE | coder_a | Primary focus is integrating social protection programmes with child nutrition in national policies; social protection programme delivery is the core instrument, with coordination as an enabling mechanism. |
| implementation_specificity | V036 | S1 | S2 | S1 | coder_a | Actions are listed as nominal recommendations (support for infrastructural adaptations, advocacy, sensitization, screening) without lexically explicit responsible actors; no "governments must" or "schools should" in the text. |
| implementation_specificity | V037 | S1 | S2 | S1 | coder_a | Primary policy action "social protection programmes should be targeted" uses passive construction without a named responsible actor; caregiver hygiene instructions name caregivers but are practice-level, not policy actions. |
| policy_instrument | V040 | I_REG | I_FISC | I_REG | coder_a | Text is about securing resources and coordination for law/policy development, monitoring and enforcement of legal measures; regulatory development and enforcement is primary, fiscal aspects support regulatory work. |
| policy_instrument | V043 | I_REG | I_ACCOUNT | I_REG | coder_a | Text addresses due process and procedural requirements in regulatory/policy development to withstand legal challenges; regulatory process compliance is primary, not outcome monitoring or accountability. |
| policy_instrument | V044 | I_REG | I_MIXED | I_MIXED | coder_b | Text lists multiple instrument types (school food policies, labelling policies, fiscal policies, health services guidelines, sodium/sugar guidelines) without prioritizing any single one; equally central. |
| implementation_specificity | V049 | S1 | S2 | S2 | coder_b | Text states "Government-led regulatory measures should be invoked, ensuring a system for monitoring...and sanctions" — government is lexically explicit as the responsible actor linked to the regulatory action. |
| policy_instrument | V054 | I_SERVICE | I_ACCOUNT | I_SERVICE | coder_a | Primary focus is implementing strategic actions across four systems (food, health, WASH, social protection) and systems-strengthening for programme delivery; accountability is a component of service delivery. |
| policy_instrument | V060 | I_PARTNER | I_ACCOUNT | I_PARTNER | coder_a | Text describes multi-stakeholder consultation with food retailers, academic experts and NGOs as the primary instrument; background statistics on unhealthy diets provide context for the partnership process. |
| policy_instrument | V069 | I_REG | I_ACCOUNT | I_REG | coder_a | Text is about law enforcement mechanisms: coordination of monitoring and enforcement, training enforcement agents, detecting and prosecuting violations; enforcement of binding law is primary. |

The absence of S3 in this balanced chunk sample cannot establish that the sampled source documents or the expert-refrozen corpus lack operational mechanisms. It may reflect chunk boundaries, conservative S3 thresholds, sampled passage types, or document genres. The study therefore reports this only as a validation-sample observation and does not infer implementation readiness.

# 5 S5. Model, boundary, and comparison diagnostics

**Supplementary Table S9A. BERTopic candidate comparison used for model selection**

| **Candidate** | **Cap** | **Discovery n** | **Clusters** | **Outliers (%)** | **NPMI** | **Diversity** | **Mean docs/cluster** | **Mean dominant-source share (%)** |
| --- | --- | --- | --- | --- | --- | --- | --- | --- |
| B17 | 40 | 730 | 14 | 11.2 | 0.190 | 0.736 | 5.1 | 67.0 |
| B14 | 40 | 730 | 11 | 19.2 | 0.191 | 0.782 | 5.2 | 64.8 |
| B09 | 40 | 730 | 16 | 7.8 | 0.193 | 0.750 | 5.1 | 67.8 |
| D15A | 15 | 320 | 6 | 10.3 | 0.194 | 0.833 | 7.5 | 31.7 |
| D15B | 15 | 320 | 6 | 12.8 | 0.195 | 0.833 | 7.0 | 32.2 |
| D20A | 20 | 414 | 5 | 1.7 | 0.146 | 0.840 | 8.0 | 30.5 |
| D20B | 20 | 414 | 5 | 8.0 | 0.118 | 0.840 | 7.2 | 33.4 |
| D25A | 25 | 494 | 3 | 4.5 | 0.096 | 0.767 | 12.0 | 24.9 |

D15A was selected by balancing coherence, diversity, outlier rate, document coverage, source concentration, and substantive interpretability. D20A and D25A reduced dominance or increased mean coverage but produced coarser five- and three-cluster solutions with markedly lower NPMI coherence; D15B had slightly higher coherence but a higher outlier rate, lower coverage, and higher dominance. The six-cluster count was not itself a selection criterion.

**Supplementary Table S9B. Model audit summary**

| **Diagnostic** | **Result** | **Interpretive restriction** |
| --- | --- | --- |
| Selected BERTopic configuration | D15A; full-corpus TF-IDF/SVD; 320 cap-15 discovery chunks; seed 42; 6 clusters | Exploratory expert-refrozen map |
| BERTopic seed stability | 0.897 mean AMI | Fixed cap-15 sample and complete-corpus representation; not external validity |
| Leave-one-document-out terms | 0.827 mean Jaccard | Omitted capped discovery chunks; retained complete-corpus TF-IDF/SVD |
| LDA stability gate | hit_max_iter_not_stable_across_seeds | Failed validation model; not corroboration |
| BERTopic-LDA correspondence | 0.390 AMI; 0.314 term Jaccard | Limited cross-model correspondence |

**Supplementary Table S10A. Full-pipeline corpus-boundary refits**

| **Scenario** | **Documents** | **Chunks** | **Clusters** | **Outlier rate** | **Penalized term similarity to baseline** |
| --- | --- | --- | --- | --- | --- |
| Prior author freeze | 19 | 1125 | 6 | 0.226 | 0.854 |
| Expert-refrozen baseline | 22 | 1259 | 6 | 0.223 | 1.000 |
| Context-expanded | 23 | 1292 | 7 | 0.317 | 0.732 |

**Supplementary Table S10B. Full-pipeline leave-dominant-source-out refits**

| **Removed source** | **Baseline cluster** | **Refit clusters** | **One-to-one match** | **Mapped top-10-term Jaccard** |
| --- | --- | --- | --- | --- |
| P032 | C2 | 6 | Yes | 0.538 |
| P017 | C4 | 3 | Yes | 0.429 |
| P031 | C5 | 5 | No | Not applicable |

**Supplementary Table S11A. Primary descriptive issuer and period comparisons**

| **Dimension** | **Cluster** | **A** | **B** | **Mean A (%)** | **Mean B (%)** | **B-A (pp)** | **95% resampling interval (pp)** | **Exact p** | **BH q** |
| --- | --- | --- | --- | --- | --- | --- | --- | --- | --- |
| Issuer | C0 | WHO | UNICEF | 12.7 | 19.8 | 7.2 | -24.7 to 36.1 | 0.762 | 0.762 |
| Issuer | C1 | WHO | UNICEF | 28.3 | 21.4 | -6.9 | -32.1 to 18.0 | 0.605 | 0.762 |
| Issuer | C3 | WHO | UNICEF | 21.6 | 12.5 | -9.1 | -28.4 to 10.3 | 0.377 | 0.762 |
| Period | C0 | 2015-2019 | 2020-2025 | 24.0 | 27.9 | 3.9 | -32.2 to 37.6 | 0.735 | 0.735 |
| Period | C1 | 2015-2019 | 2020-2025 | 35.2 | 16.2 | -18.9 | -41.1 to 3.5 | 0.122 | 0.182 |
| Period | C3 | 2015-2019 | 2020-2025 | 26.5 | 8.5 | -18.0 | -37.4 to 1.2 | 0.057 | 0.172 |

**Supplementary Table S11B. Caution-qualified descriptive issuer and period comparisons**

| **Dimension** | **Cluster** | **A** | **B** | **Mean A (%)** | **Mean B (%)** | **B-A (pp)** | **95% resampling interval (pp)** | **Exact p** | **BH q** |
| --- | --- | --- | --- | --- | --- | --- | --- | --- | --- |
| Issuer | C2 | WHO | UNICEF | 0.0 | 22.7 | 22.7 | 3.4 to 45.7 | 0.098 | 0.098 |
| Issuer | C4 | WHO | UNICEF | 37.5 | 5.0 | -32.5 | -68.9 to 0.4 | 0.058 | 0.098 |
| Period | C2 | 2015-2019 | 2020-2025 | 0.0 | 16.7 | 16.7 | 2.4 to 34.3 | 0.273 | 0.546 |
| Period | C4 | 2015-2019 | 2020-2025 | 14.3 | 17.1 | 2.8 | -31.6 to 30.4 | 0.726 | 0.726 |

**Supplementary Table S11C. Multivariate finite-corpus diagnostics for the three primary clusters**

| **Dimension** | **A** | **B** | **Clusters** | **n A** | **n B** | **Squared mean-vector distance** | **Exact p** | **Restriction** |
| --- | --- | --- | --- | --- | --- | --- | --- | --- |
| Issuer | WHO | UNICEF | 0;1;3 | 8 | 11 | 0.018 | 0.766 | Finite-corpus diagnostic; exchangeability not established |
| Period | 2015-2019 | 2020-2025 | 0;1;3 | 7 | 15 | 0.070 | 0.279 | Finite-corpus diagnostic; exchangeability not established |

Exchangeability is not established because issuer, period, genre, and document length are structurally associated and the corpus is not a probability sample. Genre-stratified permutations only partially address this dependence. P values and adjusted q values are retained as supplementary diagnostics and are not interpreted as evidence of similarity, stability, continuity, or organizational difference. The complete primary, stratified, and denominator-sensitivity outputs are preserved in tables/results/topic_issuer_comparison*.csv, topic_period_comparison*.csv, topic_comparison_sensitivity.csv, and topic_primary_omnibus_diagnostic.csv in the reproducibility archive.

# 6 S6. Independent external review

One independent professor with nutrition-policy and food-governance expertise, no involvement in corpus, model, or author coding, and no declared competing interest completed the seven-case boundary review and six-descriptor review in one author-decision-masked packet. The authors compared the completed judgments with the author-frozen corpus only after receipt. The expert changed P005, P021, and P036 to include_core and accepted all six descriptors. All three boundary changes were applied without author override, and the complete pipeline was rerun on the 22-document corpus. Descriptor decisions and the post hoc transfer audit do not establish an externally stable taxonomy; all corpus-boundary and source-concentration cautions remain. Because the author roles were masked, the expert's overall narrative used 'change' without access to the comparison baseline. Under the packet's processing rule, the seven record-level Part A decisions were controlling; comparison after receipt identified P005, P021, and P036 as the three actual changes, while P014 remained context-only.

**Supplementary Table S12. Independent expert boundary decisions**

| **Record** | **Author-frozen role** | **Expert decision** | **Active refrozen role** | **Confidence** | **Expert reason** |
| --- | --- | --- | --- | --- | --- |
| P005 | context_only | include_core | include_core | Moderate | UNICEF global-HQ, 2020. An advocacy strategy telling country offices how to press governments on food-environment regulation, monitoring and enforcement. It urges action rather than issuing recommendations, so it sits a step back from the normative instruments; on the other criteria it is a clear fit, right issuer, in window, on the food-environment domain. I kept it core, near the boundary. |
| P014 | context_only | context_only | context_only | High | UNICEF global, 2022. A communications playbook for public, policymaker and industry audiences. It's on-topic and from the right issuer, but it does messaging and framing, not normative or implementation guidance. A document can be about children's food environments and still not belong in the core corpus if it doesn't issue or implement recommendations. This one doesn't. Context-only. |
| P021 | context_only | include_core | include_core | High | Clear fit. WHO-HQ 2018 guideline on adolescent nutrition, in window, in English, operational recommendations, several directly on food environments. No reservations. |
| P025 | include_core | include_core | include_core | Moderate | UNICEF and the UN Special Rapporteur on the right to food, 2019, 28 pages. The brief frames government obligations on children's food environments through a rights lens: marketing, school food, labelling, affordability. The co-issuance with the Special Rapporteur gives it institutional weight a solo UNICEF brief would not carry. It argues a normative case rather than issuing an operational recommendation set, which keeps it at Moderate. |
| P031 | include_core | include_core | include_core | High | UNICEF global-HQ, 2022. Programming guidance on engaging the private sector, including the food industry, on maternal and child nutrition. What puts this in the core rather than context is implementation specificity: it gives programme actions, not just principles, and it names accountability mechanisms. The stance on industry engagement is UNICEF's own policy position; for boundary purposes, the document meets the core criteria. |
| P032 | include_core | include_core | include_core | High | UNICEF global-HQ, 2024. A programme framework (five entry points, ten actions) connecting child nutrition to social protection, cash transfers and affordability, aimed at programme managers and government partners. The nutrition-social-protection nexus is one of the more operationally developed areas in recent UNICEF guidance, and this document is the clearest statement of it in the corpus. |
| P036 | exclude | include_core | include_core | High | WHO-HQ, 2024, four pages on IRIS. Draft model-policy text for restricting commercial milk formula manufacturers' sponsorship of health professional associations. It's short, but it does something the other documents don't: it gives governments ready-to-adopt legislative language. That concreteness is what makes it core. |

**Supplementary Table S13. Independent expert decisions on provisional cluster descriptors**

| **Cluster** | **Final descriptor** | **Decision** | **Semantic-fit reason** | **Coverage comment on pre-amendment evidence** |
| --- | --- | --- | --- | --- |
| 0 | Food-marketing restrictions and children's rights in food environments | Accept | Accept without much thought. Marketing restriction on children's food is one of the few themes here that rests on a binding instrument: the 1981 Code, extended by successive WHA resolutions. The rights framing in the excerpts is consistent with that lineage, and P001, P006 and P007 all stay inside it. When the legal backbone is this clear, the label does not need defending. | 11 documents, 26.2% from the dominant source. Spread is fine. What holds the theme together is the legal spine, the Code and the WHA resolutions recur across the set, so the cluster should survive a change in documents. |
| 1 | Childhood overweight and obesity: epidemiological burden and global trends | Accept | These are the standard problem-statement openings that precede recommendations in almost every WHO/UNICEF guidance, 41 million overweight under-5s, 144 million stunted, burden shifting across income groups. So this cluster is really about corpus genre, not a distinct policy area. The label works as a description; I would just have the authors say that plainly in the interpretation. | 12 documents, the largest cluster, which fits: nearly every guidance document opens with this kind of framing, so the text collects into one big block. That is a genre signal, not thematic depth. I would not test it for generalizability the way I would the instrument-based clusters. |
| 2 | Nutrition-sensitive social protection and service delivery | Accept | The label is fair. The documents do connect cash transfers and household food budgets (P023) with community nutrition and programme actions (P003, P032), and the 2024 UNICEF guidance is the clearest joint statement of the two. Different instruments, but the corpus talks about them as one area, and the label follows the corpus. | 4 documents, 46.4% from one source, small and one-issuer-weighted. It reads like an emerging theme in current agency framing, not an established policy area; the authors should say so instead of presenting it as settled. |
| 3 | School, physical-activity, and healthy food environments | Accept | I accept this one, with more reservations than the others. School food and physical activity are different policy domains, different evidence bases, different levers, food standards versus curriculum mandates, and in most countries different ministries. The excerpts pull them together mainly because school is the shared setting: P002 on meal standards, P018 on physical activity in childcare. The label honestly lists all three components, so it passes as a description, but the tension is real and I would flag it rather than force a split. | 9 documents, 23.7% dominant source. The spread is fine. If the authors ever split it, each half would drop to four or five documents, so the merge is defensible on practical grounds. |
| 4 | Infant and young-child food-promotion controls | Accept | A mature regulatory regime for breast-milk substitute marketing. The digital terms are no surprise, WHA 69.9 already asked member states to cover digital channels, and P006 and P035 show it. Label is accurate. The only thought I would add: the digital dimension may deserve its own treatment eventually. | 8 documents, 50% from one source. The concentration is real but it is structural: BMS marketing guidance comes almost entirely from WHO headquarters, since that is where the Code authority sits. It is not a sampling problem. The theme is valid without spreading across issuers. |
| 5 | Business engagement and food-industry governance | Accept | Start with the concentration: 76.9% from one document decides whether this is a real theme or mostly one document's language. The terms do point to UNICEF's recent turn toward engaging the private sector rather than only regulating it (P003, P031, P037). I would push back on UPF, ultra-processed food governance is broader and more contested than business engagement, and it likely entered through the dominant document. The label describes what UNICEF is doing, which is fair; the engagement-versus-regulation tension the documents never resolve should get acknowledged somewhere. | 5 documents, 76.9% from one source. This is the one cluster where generalizability is genuinely doubtful. Take the dominant document out and the cluster may not hold together; what remains would probably be better described as conflict-of-interest management or UPF governance, both narrower. A sensitivity check without that document would settle it. |

# 7 S7. Data access and generative-AI disclosure

Public source documents are identified by landing-page URLs in Supplementary Table S2A. The prepared additional archive 1935052_Reproducibility_Package.zip contains the permitted derived CSV tables, code, hashes, and audit logs and should be mapped as an additional supplementary file in the revision portal; a repository DOI is not yet available. Source PDFs and complete copyrighted text should not be redistributed unless their licences permit it.

During the preparation and revision of this manuscript, the authors utilized OpenAI strictly as a supportive tool for English-language copy-editing and basic syntax assistance during code development. All analytical scripts, reproducibility checks, and data-consistency validations were conceptualized, directed, and rigorously verified by the authors. No generative AI image tools were used. All five figures and all tables are deterministic, data-derived outputs programmed and generated entirely through the authors' own analytical scripts using audited research data. The intellectual execution of this study remains entirely human. No AI system was involved in any substantive or decision-making processes, including literature screening, qualitative coding, corpus boundary setting, adjudication, data analysis, or the interpretation of findings. All author-stage decisions were made independently by the authors, supplemented only by the masked judgments of one independent human expert (as detailed in Sections 2.3 and 2.7). The authors critically reviewed all AI-assisted language and code outputs, and assume full and ultimate responsibility for the originality, accuracy, and integrity of the final manuscript.
